# Considering epitopes conservity in targeting SARS-CoV-2 mutations in variants: a novel immunoinformatics approach to vaccine design

**DOI:** 10.1038/s41598-022-18152-5

**Published:** 2022-08-18

**Authors:** Mohammad Aref Bagherzadeh, Mohammad Izadi, Kazem Baesi, Mirza Ali Mofazzal Jahromi, Majid Pirestani

**Affiliations:** 1 Student Research Committee, Jahrom University of Medical Sciences, Jahrom, Iran; 2 Department of Virology, Pasteur Institute of Iran, Tehran, Iran; 3 Zoonoses Research Center, Jahrom University of Medical Sciences, Jahrom, Iran; 4 Department of Immunology, School of Medicine, Jahrom University of Medical Sciences, Jahrom, Iran; 5 Department of Advanced Medical Sciences & Technologies, School of Medicine, Jahrom University of Medical Sciences, Jahrom, Iran; 6 Parasitology and Entomology Department, Faculty of Medical Sciences, Tarbiat Modares University, Tehran, Iran

**Keywords:** Genome informatics, Peptide vaccines, Genomic instability, Target identification

## Abstract

Severe acute respiratory syndrome coronavirus-2 (SARS-CoV-2) has gained mutations at an alarming rate in the past years. Developing mutations can increase the virus's pathogenicity and virulence; reduce the efficacy of vaccines, antibodies neutralization, and even challenge adaptive immunity. So, it is essential to identify conserved epitopes (with fewer mutations) in different variants with appropriate antigenicity to target the variants by an appropriate vaccine design. Yet as, 3369 SARS-CoV-2 genomes were collected from global initiative on sharing avian flu data. Then, mutations in the immunodominant regions (IDRs), immune epitope database (IEDB) epitopes, and also predicted epitopes were calculated. In the following, epitopes conservity score against the total number of events (mutations) and the number of mutated sites in each epitope was weighted by Shannon entropy and then calculated by the Technique for Order of Preference by Similarity to Ideal Solution (TOPSIS). Based on the TOPSIS conservity score and antigenicity score, the epitopes were plotted. The result demonstrates that almost all epitopes and IDRs with various lengths have gained different numbers of mutations in dissimilar sites. Herein, our two-step calculation for conservity recommends only 8 IDRs, 14 IEDB epitopes, and 10 predicted epitopes among all epitopes. The selected ones have higher conservity and higher immunogenicity. This method is an open-source multi-criteria decision-making platform, which provides a scientific approach to selecting epitopes with appropriate conservity and immunogenicity; against ever-changing viruses.

## Introduction

About 2 years have passed since the first case of new coronavirus disease-19 (COVID-19) has been identified in Wuhan, China. Severe acute respiratory syndrome coronavirus 2 (SARS-CoV-2), the notorious claimant behind COVID-19, has spread all around the world, soon after confirmation of the first cases in Wuhan, which resulted in a global health problem till now^1–3^. On 11 March 2020, the World Health Organization (WHO) announced this worldwide health problem as a new pandemic. Although the pandemic is quite young, now our knowledge about the etiology, pathogenesis, and treatment of COVID-19 has improved^4–6^. SARS-CoV-2, the new member of the Coronaviridae family (Betacoronavirus), has improved its ability to survive and preserve its generation^5^. Compared to other Coronaviridae members, higher virulence besides significant pathogenicity may justify this unprecedented pandemic^7^.

According to the latest report of WHO on 2/13/2022, so far 404,910,528 people in the world have been infected with the SARS-CoV-2, and 5,783,776 have died from COVID-19^1^. COVID-19 clinical presentations range from non-symptomatic infection to severe acute respiratory distress syndrome (ARDS) and even death, but it is best known for its flu-like symptoms (cough, low-grade fever, chills, myalgia, sweating, and fatigue), as well as pneumonia^8,9^. By passing time, observations in clinical practice declared other organ involvement like the gastrointestinal and central nervous system in COVID-19 that just complicated the issue^10,11^. The impact of COVID-19 on individuals and also public health although is drastic, it is far to be understood. So far, several vaccines with different platforms have been approved by Food and Drug Administration (FDA) or local authorities^12^. Vaccination and adhering to safety protocols (reducing human-to-human transmission through monitoring, social distancing, and hygienic principles) are promising possible options^13,14^.

Therefore, the vaccine-mediated immunity (vaccination) of the population seems to be even more important in the current situation of the COVID-19 pandemic, where SARS-CoV-2 spreads rapidly and there are therapeutic challenges in the management of patients suffering from COVID-19. In another hand, the naturally acquired immunity (NAI) could not prevent the spread of the SARS-CoV-2^15^. So, the importance of developing vaccines to target SARS-CoV-2 during this particular circumstance is undeniable^16^. Many noteworthy considerable efforts have been made in this field around the world. Till now, different approaches have been administrated for COVID-19 vaccine design, such as mRNA-based vaccines (Comirnaty (BNT162b2 or Pfizer, BioNTech) and Moderna), adenovirus vaccine (AstraZeneca), recombinant adenovirus vaccine (Sputnik V), non-replicating viral vector (Janssen (JNJ)), inactivated vaccine (CoronaVac), peptide vaccine (EpiVacCorona), nanoparticle vaccine (NVX-CoV2373), and DNA vaccine (plasmid) (ZyCoV-D). Meanwhile, some have international approval for global use, some have limited licenses in some countries, and a large number of vaccines are under the clinical trials and development phase (more information on vaccine tracker-NY)^17^. According to WHO, as of 2/13/2021, 10,095,615,243 vaccine doses have been administrated in the world^1^.

As vaccines are being developed in the laboratories and vaccination is under process, the novel coronavirus is cleverly changing and mutating in nature. So far, lots of mutations have been identified in the SARS-CoV-2 genome, which resulted in new specific variants. Potentially developing mutations can result in structural changes in key proteins involved in the pathogenesis and spread of the virus. The frequent and rapid genetic mutations and the consequent changes in SARS-CoV-2 implicate the necessity of a method for naming new variants. As expected, new mutations in the target sequences of vaccines could decrease vaccines efficacy against new variants, besides increasing pathogenicity, transmission, and virulence, a point that has also been shown in studies^18–20^. According to the United States centers for disease control and prevention (CDC), variant of interest (VOI) stands for a variant with specific genetic markers, which potentiate changes to receptor binding, decreased neutralization by antibodies generated against vaccination, and some other clinical issues in diagnosis and treatment. In another hand, variant of high consequence (VOHC) indicates a variant with significantly reduced effectiveness of prevention measures or medical countermeasures relative to previously circulating variants, in literature with acceptable evidence^21^.

The CDC illustrated that B.1.617.2 and AY lineages (Delta), B.1.1.529 (Omicron), are categorized as current variant of concern (VOC), which means they are associated with reduced neutralizing antibodies titer in the convalescent and post-vaccination sera besides increased transmissibility and more severe disease^22–24^.

It shows regardless of various approaches to vaccine design; the vaccine target needs to be selected precisely. As SARS-CoV-2 evolves spontaneously, two major points should be considered in selected parts’ properties; first, the selected part must have an acceptable antigenicity to induce the significant immune response, second, it must be conserved during mutations in order to obtain proper coverage against different present variants. Also, it helps to maintain its efficacy against new variants. But on the other side of the coin, it might be more tremendous. Deployment of data and tools to understand the manner of SARS-CoV-2 mutations and simulation of the immune response against new variants seem to be a reliable way to deal with this issue^25^.

On the other hand, different bioinformatics approaches have been utilized for finding potential drugs^26–28^, designing vaccines^29,30^, and finding the concept behind COVID-19 pathogenesis^31,32^. Many complex biochemical processes that have to be spent in the laboratory with a lot of time and money can be modeled and predicted^33,34^. For example, finding herbal drugs’ interaction with SARS-CoV-2 proteins^33,35,36^, predicting immunogen and antigen epitopes from SARS-CoV-2 proteins^37,38^, and finding new potential pathways in COVID-19 progression and pathogenicity^32,39^ and etcetera are all available through different bioinformatics techniques.

In this study, first, we demonstrated how investigated immune-dominant parts of SARS-CoV-2 proteins have mutated significantly. Furthermore, we presented evidence of alterations the spike (S), membrane (M), nucleocapsid (N), and envelope (E) proteins of SARS-CoV-2, which are discussed in peer reviews from the immune epitope database (IEDB)^40–42^. Because of the necessity of finding the appropriate target for vaccines, we wondered how an algorithm could propose an epitope with significant antigenicity besides being adequately conserved.

Here, we predicted B cell, helper T lymphocyte (HTL), and cytotoxic T lymphocyte (CTL) epitopes and then aligned them to their reference sequence to find mutations in each epitope. Then we scored their events (mutations) with the Shannon entropy and Technique for Order of Preference by Similarity to Ideal Solution (TOPSIS) method to quantify their conserved manner. In the setting of this algorithm, the final output still included dozens of different types of epitopes from different proteins making the decision hard to select the best epitope. To select ones with superiority, epitopes were illustrated in a plot of antigenicity score–conserved manner, and then epitopes were undergone illustrated two-dimensional comparison, which allows facile and confident selection of epitopes among a large volume of data. In this logical analysis, we tried to clarify the importance of the conserved property of epitopes along with the immunogenicity of the selected parts of SARS-CoV-2 proteins for the vaccine design (study design—sup. Diagram 1).

Herein, we provided a scientific and practical approach selecting epitopes with appropriate conserved and immunogenicity properties (whether in predicted epitopes or real ones from databases or peer reviews), with the hope that this protocol will aid in the development of methods to design vaccines against ever-changing viruses such as SARS-CoV-2, human immunodeficiency virus (HIV) and so on.

## Results

### Background

Genetic mutations elsewhere in the SARS-CoV-2 genome resulted in sophisticated conditions both in the virus virulence and pathogenicity and also in the host's immune system response by repeated infections with other variants that rechallenge adaptive immunity response^20,43^. Perhaps, the most important issue in dealing with the coronavirus vaccine design is its genetic variation^44–46^. Not only does it affects the vaccine design, but also it challenged the immune system response and treatment^46,47^. This issue, typicality is not a new concern, as we have seen this challenge in other viruses (such as, Influenza virus, Herpes, Zika, other coronaviridea members, and etc.)^48–50^. The most prominent example of these is HIV, where all these problems (in the immune system response against the virus, clinical presentation and management, and also vaccine design) are perspicuous^51,52^. As previously predicted, this problem grew rapidly with the introduction of VOC (B1.1.7, 501Y.V2, etc.), which prompted the WHO to quickly propose a system for naming new variants of the virus. We could be optimistic that by developing a strategy to deal with RNA virus mutations, the main burden against these viruses will be removed.

### Finding mutations in SARS-CoV-2 variants

**Figure 1:**
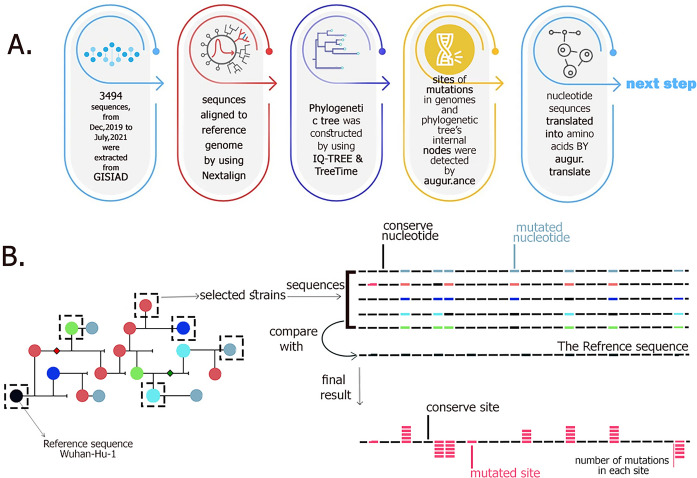
Schematic illustration of 5 steps to find the site of mutations and number of mutations. As illustrated in panel (**A**) number of mutations in each site of the SARS-CoV-2 genome was identified through 5 steps for the next step (finding mutations in different sites of epitopes and IDRs and quantifying their conservity). In panel (**B**), the identification of mutations and sites with comparing with the reference sequence (3rd and 4th steps) is illustrated schematically. In this example we have chosen 5 new variant sequences and compared them with the reference sequence in order to find the site of mutations and number of events in SARS-CoV-2 genome.

**Figure 2:**
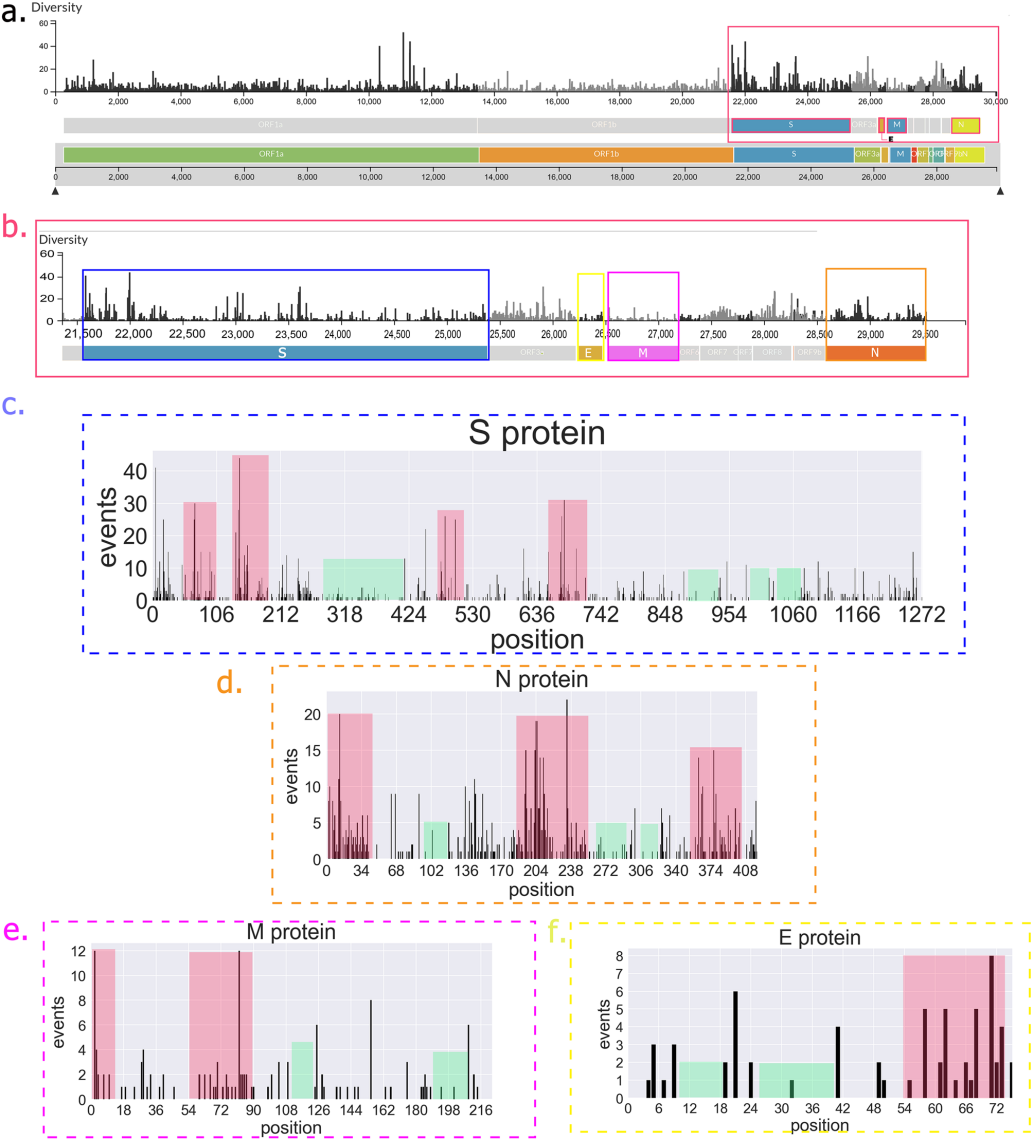
Mutations (events) in SARS-CoV-2 surface proteins (S, N, M, and E proteins) based on changes in their amino acid sequences. All events have been recorded on SARS-CoV-2 proteins illustrated in the first diagram (panel **a**). We focused on 4 main surface protein changes (the red box, panel **b**). In panel c (dash lined blue box), S protein events are illustrated. Qualitatively, we can see some red areas that have a higher number of mutations in their sequences (or high density of mutations), in comparison with green areas with the lower number of mutations in their sequences. All these changes and this qualitative view (red and green areas) are also presented for N, M, and E proteins in panels d, e, and f respectively.

In order to detect mutations in the SARS-CoV-2 genome, 3369 sequences, from Dec 2019 to July 2021 were extracted from the global initiative on sharing avian flu data (GISAID) database (Fig. 1). Then, the sequences were aligned to the reference genome (hCoV-19/Wuhan/Hu-1/2019 (NC_045512.2)) using *Nextalign*. According to literature and the National Center for Biotechnology Information (NCBI), NC_045512.2 was considered as the reference sequence^5,53–55^. After that, sequences that were incomplete or much shorter than the reference sequence [having more than 3000 (non-template nucleotide) Ns and gaps (‘–’)] were excluded (Sup. Algorithm 1). The phylogenetic tree was constructed by using *IQ-TREE & TreeTime* (phylogenic tree—S1)^56^. Afterward, using the *augur ancestral module*, sites of mutations in genomes and phylogenetic tree’s internal nodes were detected, as well as in the following translated into amino acids by the *augur translate module*. In the following, the number of mutations in each site was calculated with Python programming (Figs. 1 and 2). In this way, we could illustrate mutations in each region of the genome for each variant or total variation in each protein (Figs. 2 and S2).

In this study, we mainly focused on surface proteins that are mostly targeted by the immune system and vaccine design. In fact, events (alteration in the amino acid sequence of proteins) of the spike (S), membrane (M), nucleocapsid (N), and envelope (E) proteins have been magnified and illustrated (Fig. 2). As demonstrated in Sup. Table 3, about 30% of the amino acid positions of S, E, and M proteins and about half of the amino acid positions of N protein have been mutated or at least have one event. A brief look at the charts (Fig. 2) reveals some areas with a high number of events (red area) and some areas with fewer mutations (green area). For example, in the case of S protein, in about the first 220 amino acid sites, there is a high density of events besides the great number of mutations in several sites. In another hand, we can see areas with a low number of mutations that seem to be more conserving (green area). However, this type of categorization into green and red areas has been done upon qualitative decision, but it is clear enough to demonstrate the obvious difference in terms of conservity among sites of each protein. In the following, we tried to calculate this qualitative difference into quantitative form for better decision-making. Before addressing the next step, here we highlighted the concerns with these differences (green and red areas).

**Figure 3:**
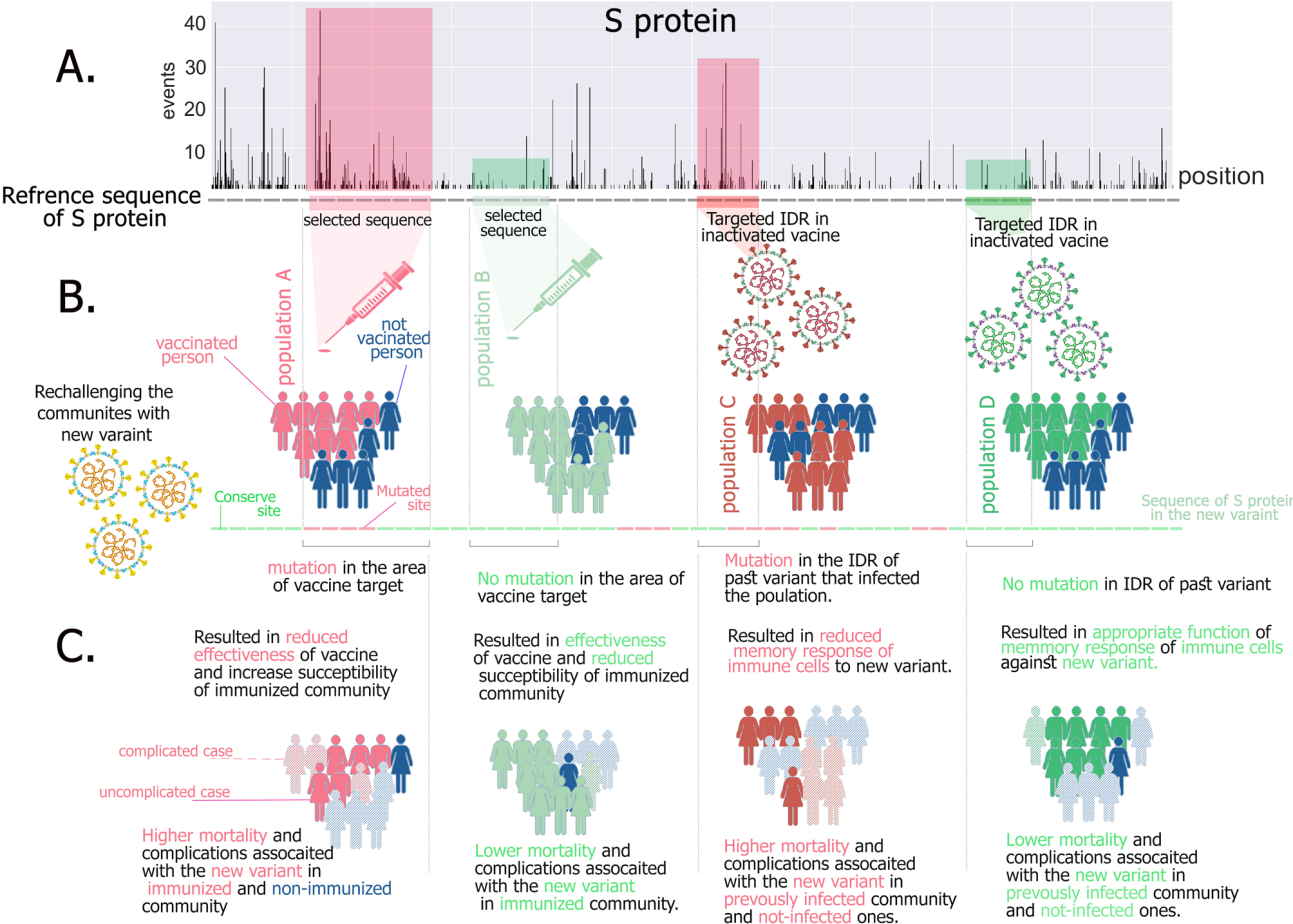
Different population with different exposure and vaccination. Panel (**A**) presents a mutational diagram of S protein with conserved and mutated sites and areas as demonstrated before. In panel (**B**) a schematic view of the S protein sequence with mutations and different regions with the qualitative view is illustrated. Different populations with varying types of immunization from various parts of the S protein sequence are presented. In panel (**C**), a rechallenge of all communities with a new variant is shown and the outcome of different sites of S protein that had been used for immunization is illustrated schematically. Blue dummies in the figure represent the not vaccinated population and the pale ones represent dead people in the community. Painted dummies in all colors except dark blue represent past infection or vaccination.

Suppose the population A (Fig. 3), where a significant number of people have been vaccinated with the vaccine A. At the time of designing this vaccine, there has been little data available about SARS-CoV-2 mutations. In fact, the epitope selected from the reference sequence of SARS-CoV-2 for designing vaccine A is accidentally located in the red area of the S protein, although it has significant immunogenicity. In another case, consider population B, where a significant number of people have been vaccinated with vaccine B. The epitope selected from the reference sequence of SARS-CoV-2 for designing vaccine B is accidentally located in the green area of the S protein. In population C a majority of communities have been infected with the strain C of SARS-CoV-2 previously.

This strain has an immunodominant region (IDR) in the red area of the S protein. In another hand, consider population D, where a majority of its community has been infected with the strain D of SARS-CoV-2, previously. Strain D also has one IDR in the green area of the S protein. In the dissemination and infection of all 4 populations with a new strain of SARS-CoV-2, which shares lots of mutations in all areas mainly in red areas; the difference will be more noticeable. In this example, it is somewhat obvious that populations A and D are more affected by the new strain of the virus. These populations would have higher infected patients in hospitals and higher mortality rate. With this view of the epidemic and considering the ever-changing virus, it is possible to better analyze that population is more affected by the new strains. With this in mind, it is also possible to reduce the burden of the disease on the community at risk by properly planning vaccination with the appropriate vaccine against the new species spreading. As a large number of mutations are found in different regions of the virus surface proteins, selecting areas that are less mutated is difficult to target with vaccination. But if such an area can be detected to target with the vaccination, it can be hoped that the designed vaccine will work well against the variants so far. In addition, this vaccine will also be less likely to be targeted by new mutations in the virus.

In the following, we tried to target regions in the virus genome that have appropriate immunogenicity besides acceptable conservity.

### Investigating mutations within IDRs of SARS-CoV-2

The immune response to SARS-CoV-2 has been investigated in past years. As of May 2021, 3330 SARS-CoV2 linear epitopes were available on IEDB as they have been reported in the peer-reviewed literature. About 2/3th of them (2203 epitopes) were from S, M, N, and E proteins. These epitopes are derived from the SARS-CoV-2 genome and they have been investigated in the laboratory if they could stimulate the immune response. In this study, we have focused on S, M, N, and E protein derived B cell, HTL, and CTL epitopes (Sup. file 1).

**Figure 4:**
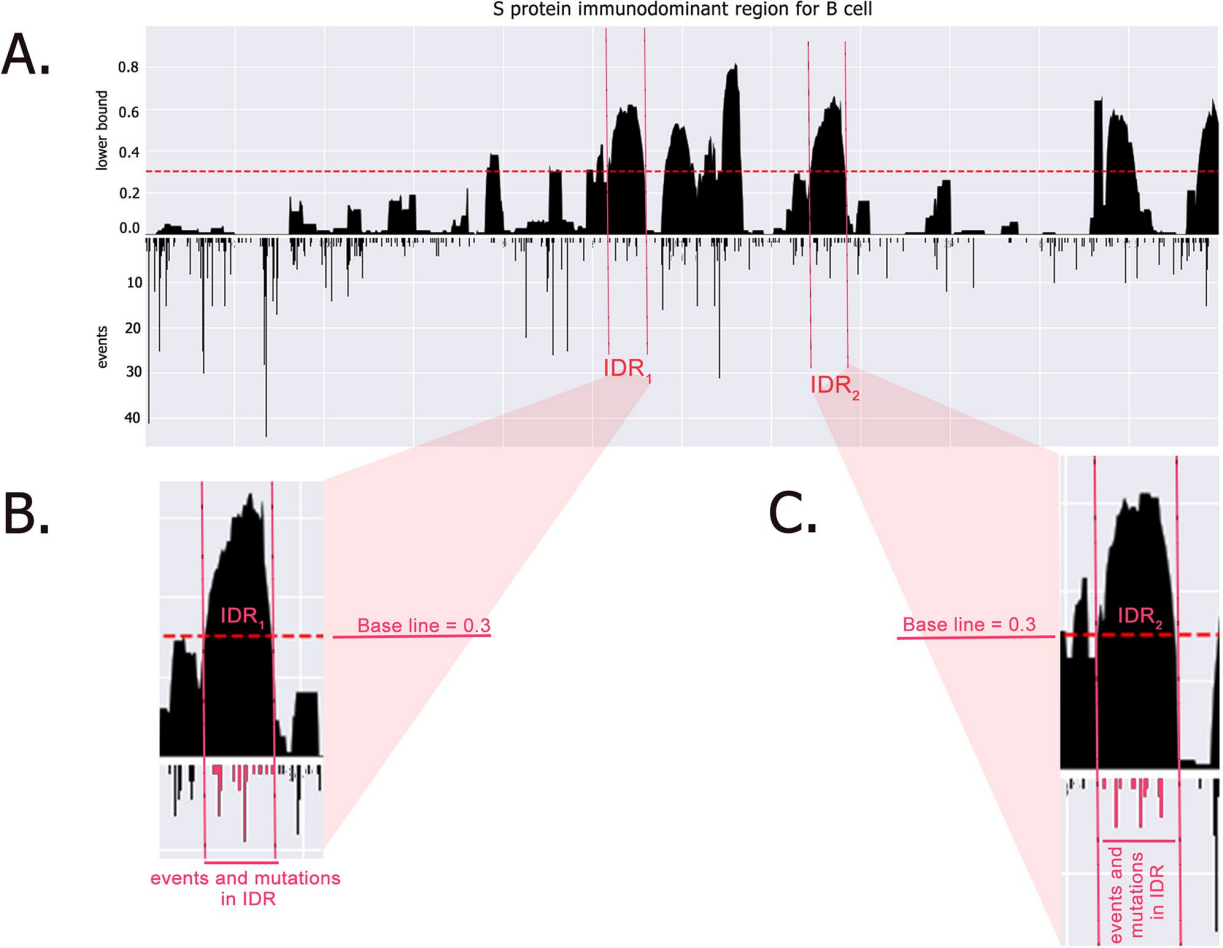
Example of IDRs of S protein for B cell response and events in their IDRs. In panel (**A**), two examples of IDRs (panel **B**,**C**) are magnified and events in their site (vertical bars) are shown. This is a schematic view of understanding how we calculated the number of mutations in IDRs. For example, in panel (**B**) we can see an IDR with a length of about 30 amino acids with many mutations at its site.

So, we have extracted B cell, HTL, and CTL epitopes from S, M, N, and E proteins from IEDB. Here is to note that this search result is restricted to epitopes with recognition by major histocompatibility complex (MHC) class I and class II molecules. Then, extracted epitopes were mapped back to a SARS-CoV-2 reference sequence^53^ using the IEDB’s immunobrowser tool^57^. This tool helps to identify the IDRs by considering their response frequency (RF) score or lower bound in the diagram (where the RF score was RF ≥ 0.3 considered as IDR) (Fig. 4). As all available records aligned along the reference sequence, the RF score was calculated by the positivity rate (positive response noted) divided by the total number of records (number of independent assays) (see the following equation)^58^.

$${\text{RF}} = {\text{ Positive response rate}}/{\text{Total number of records}}$$

Our result demonstrated several IDRs in each protein. S protein has about 12 IDRs in terms of B cell epitopes and 5 IDRs in terms of HTL epitopes (Sup. Figs. 2 and 3). The numbers of IDRs and events in IDRs of each protein are summarized in Sup. Table 2. By merging 1st section data and this section, variations in IDRs become more prominent. As there are considerable numbers of mutations in IDRs (Fig. 4), significant immune response alteration cannot be out of mind. Because these areas (IDRs) are most likely to be identified by the immune system and result in an appropriate immune response stimulation against the virus, IDRs are suitable targets for vaccine design. And also, mutations in such areas, as illustrated in Fig. 4 and Sup. Tables 1 and 2, could be a possible challenge for memory cell response.

This means even a person, who has previously been infected with the virus or has been vaccinated is still prone to be infected by new variants of SARS-CoV-2 like population C. This issue can explain to some members of VOCs that they are associated with a decrease in the efficacy of vaccines and monoclonal antibodies (such as omicron and delta variants) in the neutralization and treatment of patients suffering from COVID-19^23,59–64^.

**Figure 5:**
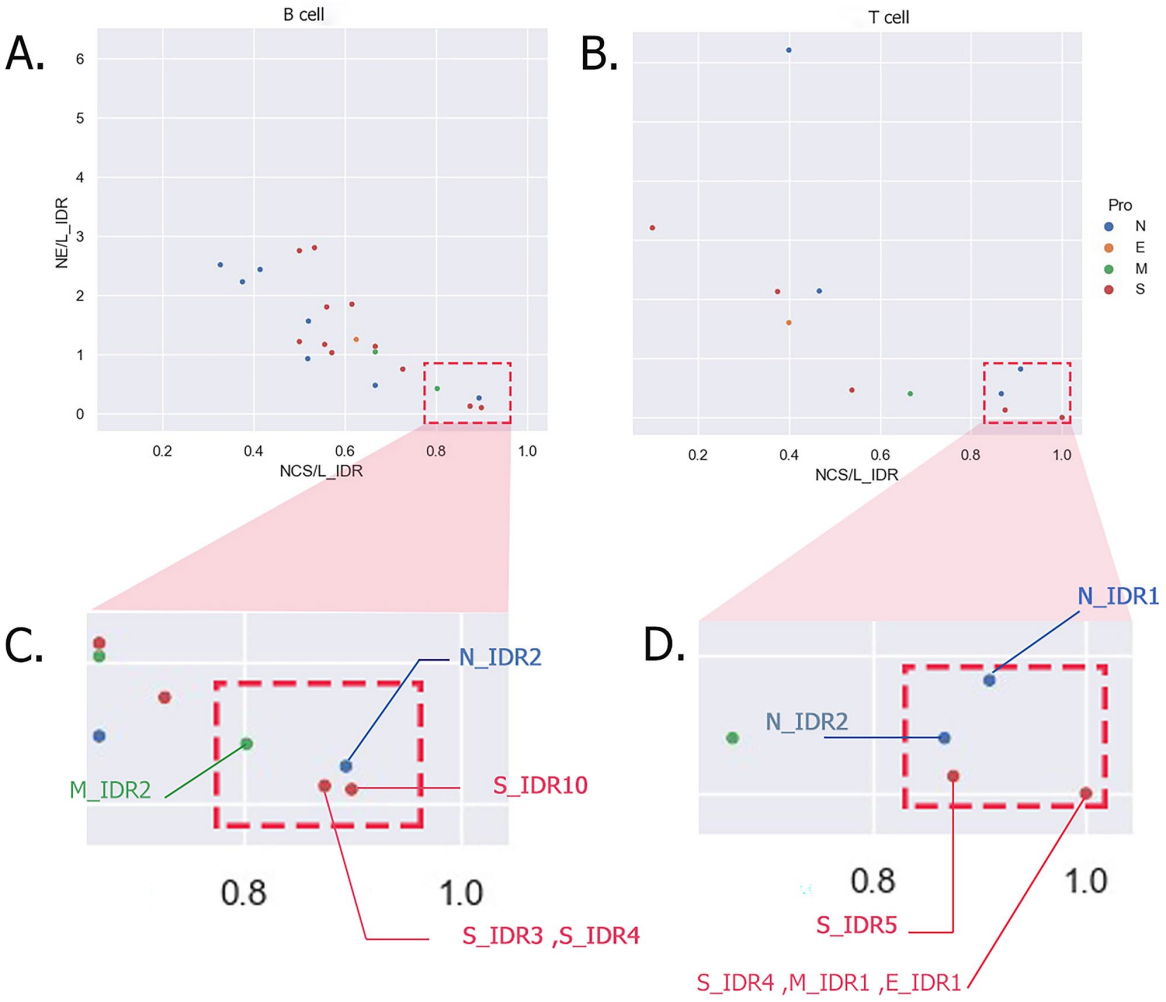
IDR plot. *NE* number of events, *L_IDR* length of IDR, *NCS* number of conserve sites. In panel (**A**) T cell IDRs are plotted with their number of events, their length and the number of conserve sites. As the number of conserved sites increases and the number of events decreases (Right Lower Quadrant), the IDR is more conserved in variants. Several T cell IDRs are selected as the most conserved IDRs (magnified in panel (**C**)). In panel (**B**) B cell IDRs are plotted with the same parameters. Several B cell IDRs are selected as the most conserved B cell IDRs (magnified in panel (**D**)). Here is to note that one dot in the plot can represent several IDRs that have the same normalized conserve sites and events.

Here IDRs are plotted, to make a quantitative comparison among all IDRs in terms of several mutated sites and events. As a number of events and mutated sites are not comparable due to the different lengths of IDRs, we normalized these parameters by dividing them using the length of each IDR (Fig. 5).

As illustrated in Fig. 5, IDRs are compared in plots in terms of their conservity. There are several B cell IDRs (Fig. 5, Left graph) in S, M, and N proteins that have appropriate conservity (red box). In T cell IDRs (Fig. 5, Right graph) there are 6 IDRs seem to be conserved. This means that these selected IDRs in the plot contain epitopes with higher conservity. Thereupon, selecting epitopes from these areas in the SARS-CoV-2 genome for further vaccine design seems to be more effective against variants discovered thus far. These IDRs sequences and locations are available in the supplement (Sup. file 1).

As mentioned, each IDR includes several epitopes. According to significant mutations in IDRs, we wondered if past reported epitopes were affected by mutations in new variants of SARS-CoV-2. In the next section, we try to understand how different mutations in the SARS-CoV-2 genome affected IEDB suggested epitopes in past laboratory research and also predicted ones.

### Significant mutations in SARS-CoV-2 IEDB epitopes

**Figure 6:**
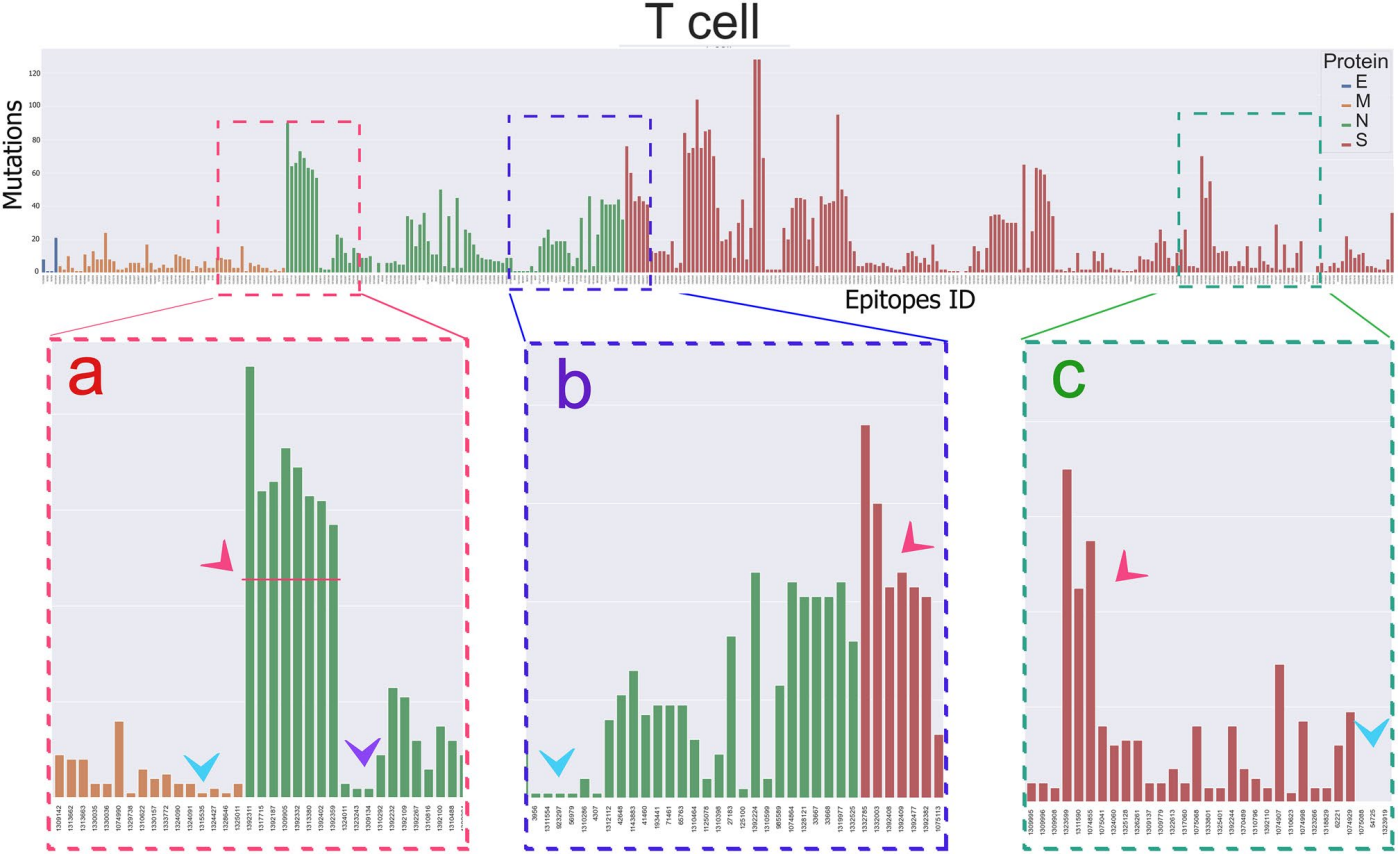
Mutational diagram (MD); Illustration of IEDB epitopes and their mutations. In this figure number of mutations is illustrated in vertical bars and epitopes ID is under each bar in horizontal axis. Panel (**a**–**c**) magnifies 3 different parts of MD. For example, in panel (**a**) there are epitopes from M protein (orange bars) that have a small number of mutations (sky blue arrows head) besides, epitopes from N protein (green bars) with the high number of mutations (pink arrows head). Purple arrows head point to sites with a moderate amount of mutations.

As mentioned before, we mapped all mutations from known variants to SARS-CoV-2 M, N, E, and S protein sequences (Fig. 4). Also, we have extracted B cell, HTL, and CTL epitopes of these proteins from IEDB. Then epitopes were aligned to their reference sequence, in order to map the mutational diagram (MD) of each protein. So, we could find out where each epitope is located in its protein sequence and how this located part has undergone alterations by mutations (Sup. Fig. 4 and Fig. 6).

A list of IEDB epitopes and their identity code is available in the supplement (Sup. file 2). As demonstrated, almost all investigated IEDB epitopes of proteins have undergone mutational changes (Fig. 6 and Sup. file 3). But the other point of view, which attracts attention, is the difference in the number of mutations in different epitopes. In Fig. 6, there are epitopes with a high number of mutations (Fig. 6a red arrows head) besides, epitopes with a low number of mutations (Fig. 6b blue arrows head) or even absolutely conserved ones (Fig. 6c blue arrows head).

The results show that almost all epitopes that have been studied in the laboratory over the past years have changed in the same period. As these epitopes were evaluated and utilized for vaccine design, a reduction in the effectiveness of vaccines is very likely. As well, it could be an important alarm for the international community and clarify the necessity of research on new variants for vaccine design development. It may also be a better option to consider faster and more efficient approaches in vaccine design, such as mRNA vaccines.

Since most epitopes are mutated in the case of SARS-CoV-2, it makes sense to choose epitopes with minimal changes (fewer mutations) and maximum stability (higher conservity). While considering epitopes conservity, maintaining immunogenicity properties also should be noted. Given that, choosing the best epitope should be considered several factors that make the choice more difficult. This makes the issue more complicated than previous approaches (where just immunologic properties were important). Hence, we kept our research on new variants with a focus on how to choose the best epitope for further investigations and application in vaccine design and development.

### Plotting events against immunologic properties of IEDB epitopes

We choose RF representative IEDB epitopes immunologic property as X-axis value. Besides, all mutations (without considering the site of mutation) in each epitope have been selected as events of the Y-axis. In this way Epitope Event—RF Plot (EVRP) depicts past events to each epitope beside their immunologic property (Sup. Fig. 5). This means epitopes events can be studied retrospectively in the case of variants and mutations.

As can be seen in (Sup. Fig. 5), all epitopes are arranged according to the two factors in the EVRP. For vaccine design, epitopes are desirable that have good immunogenicity along with a low number of mutations. So, the lower right quadrant of this chart simply shows the best epitopes. It is also possible to easily compare two or more separate epitopes in this chart in terms of both factors. EVRP also shows that a large number of epitopes detected and tested in the laboratory are either weakly immunogenic (especially in the case of T-cell epitopes) or have been affected by dozens of mutations. Only a handful of them is suitable for further research in vaccine design.

In past, we had to consider thresholds for each parameter and just omit data under threshold value and then address the remaining data for evaluation with another parameter. But in the case of EVRP two parameters were illustrated simultaneously for each epitope, which facilitates comparison between epitopes. This feature allows us to choose the best among a large number of epitopes from different proteins by considering two characteristics.

Also, in the past approach, it was difficult to compare a large number of epitopes. Herein, comparing several epitopes is easy. Although EVRP is a better tool for comparison, it cannot generate an accurate valuation for each epitope based on the site of the mutation and the number of the mutations.

For instance, consider a and b epitopes with lengths of 10 amino acids (Sup. Fig. 6). Epitope a gained 10 mutations in 2 sites and b epitope gain 10 mutations in 5 sites. EVRP just illustrates them in the same position in the diagram, although epitope b’s conservity is lesser than epitope a. As non-conserved sites increase in epitopes, the probability to gain further mutations rises. Thereupon, it is necessary to properly weigh each of the parameters to make epitopes comparable.

In the past sample (epitopes a and b) we compared epitopes of equal length, but in reality, we face epitopes with different lengths from different proteins. These issues make analogy difficult. So, we utilized another method for epitope evaluation to differentiate these cases.

TOPSIS is a multi-criteria decision-making approach, suitable in situations where several factors influence the decision. This technique was developed in the late twentieth century by Ching-Lai Hwang and Yoon^65^. In addition, TOPSIS is a concept that the chosen alternative (parameters) should have the shortest geometric distance from the positive ideal solution (PIS) and the longest geometric distance from the negative ideal solution (NIS).

Here, we used TOPSIS to quantify and Shannon entropy to weight events of epitopes logically considering two parameters: the sites of mutations and, the number of mutations in each site.

**Figure 7:**
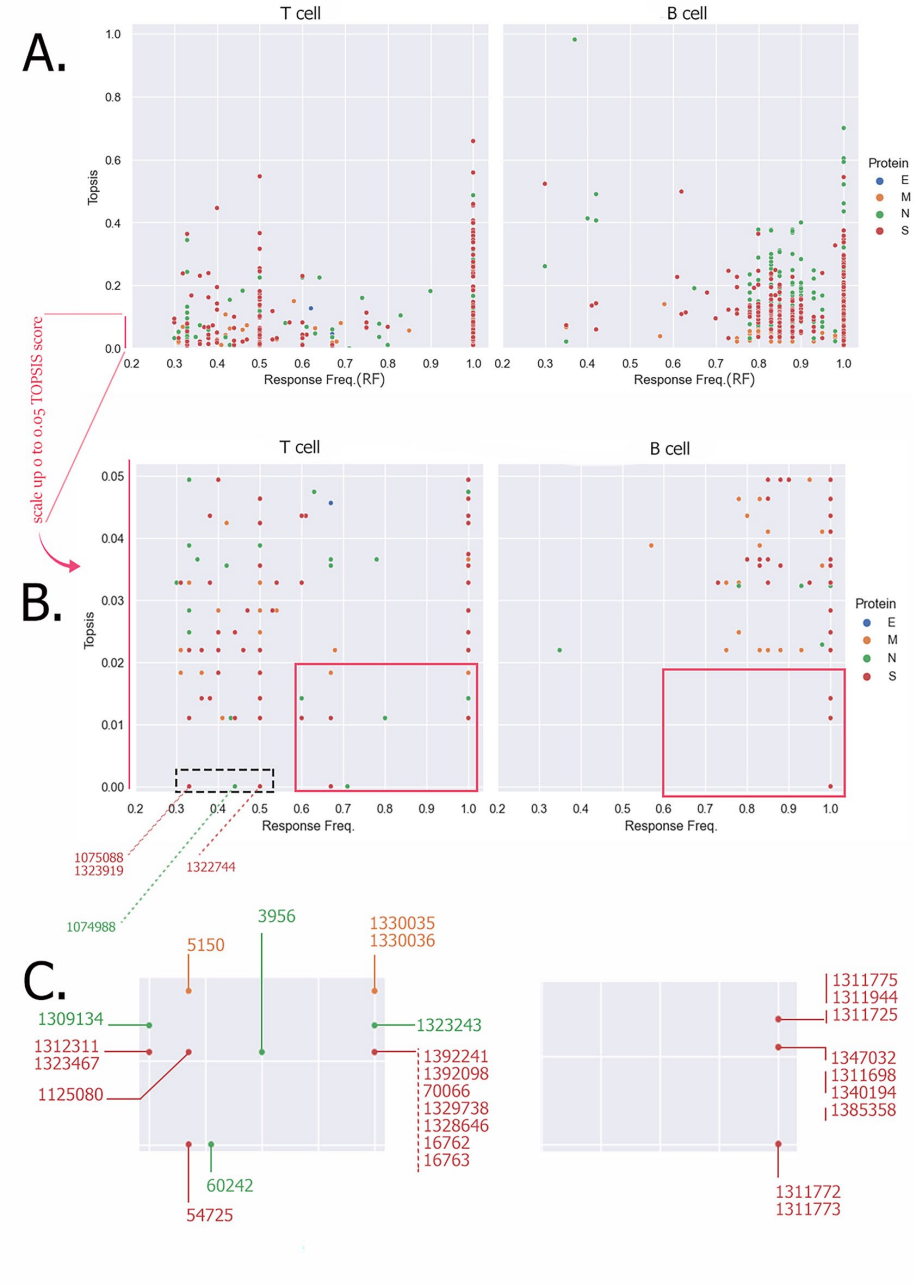
ERTP illustrates IEDB B cell and T cell epitopes immunologic property (by RF) along with conservity by TOPSIS scoring. In Panel (**A**) a logical comparison between IEDB epitopes has made through TOPSIS score (vertical axis) and Response Frequency (horizental axis). It helps to understand difference between IEDB epitopes with the same number of events but a distinct number of mutated sites. Panel (**B**) scales up vertical axis (TOPSIS score) for better illustration of position of different dots in the diagram. Right lower quadrant presents epitopes with higher immunologic properties besides higher conservity (the red boxes). In panel (**C**) red boxes are magnified and ID of each epitope (dot) is noted. It should be note that some dots represent for several epitopes with the same RF and TOPSIS scoring.

So, we kept RF on X-axis but we changed the Y-axis value to the TOPSIS score of mutations for epitopes. Epitope RF–TOPSIS Plot (ERTP) is designed to solve previous problems and facilitate comparison among epitopes while considering several factors (Fig. 7). By rearranging the epitopes based on the factors weighed down with TOPSIS, it is possible to make a more accurate comparison between them. The ERTP diagram clearly shows how the epitopes examined so far have been affected by mutations at different points in their sequence.

It should be noted that the lower the TOPSIS score, the more conservative the epitope is. Epitopes are worth more research in the laboratory or clinic, which are in a more appropriate area in the chart than others. We are currently facing a pandemic of a constantly evolving virus; we must use the best of the available vaccines based on the strain spreading in each region. Vaccines made from the inactivated pathogen should be screened for IDRs in the same system as introduced, and other vaccines targeting a specific epitope should be evaluated by the same system. As mentioned, mutations are inevitable in IDRs and epitopes in the case of SARS-CoV-2. But to counter the new variants and especially VOC, we can administrate immunogenic regions that have been less mutated so far for vaccine design.

So far, we have discussed the epitopes that have been discovered and tested in the laboratory. Herein, we wonder if this system could be effective to evaluate the new predicted epitopes by immunoinformatic databases. Predicting and evaluating epitopes with ERTP can facilitate designing new vaccines against the spreading variants. This not only helps to produce more effective vaccines but can also greatly reduce the cost of vaccine production and save time.

At this point we decided to run the ERTP method on SARS-CoV-2 predicted epitopes as well. To find epitopes those, despite high immunogenicity, are less mutated between different variants (conserve among variants so far).

### Prediction and assessment of SARS-CoV-2 epitopes

#### CTL epitopes

By using the NetCTL v1.2 server a total of 700 CTL epitopes were predicted for 12 MHC class I supertypes for M, N, E, and S proteins. Then their immunogenicity was assessed by IEDB class I immunogenicity tool and 400 of them had been predicted to be immunogens. Out of these 400 epitopes, 233 non-toxic (by ToxinPred server) and non-allergenic (by AllerTOP 2.0 server) ones were predicted and selected for further evaluation. In the end, 114 immunogenic, non-toxic, and non-allergenic epitopes were analyzed by the Vaxijen server^66,67^ in the case of antigenicity and their scores were recorded for plotting as the X-axis value (Sup. file 4).

#### HTL epitopes

94 probable HTL epitopes were predicted for S, M, N, and E proteins by IEDB MHC class II allele binding prediction tool (percentile rank ≤ 0.25)^68,69^. In the case of HTL predicted epitopes, 64 epitopes were non-allergenic and nontoxic by AllerTOP 2.0^70^ and ToxinPred^71,72^ server, respectively. As induction of interferon (IFN)-γ, interleukin (IL)-4, and IL-10 secretion by these epitopes play a pivotal role in regulating immune response, HTL predicted epitopes were analyzed for these properties. Using the IFN epitope^73^, IL4pred^74^, and IL10pred^75^ servers, 5 epitopes were left for the next step. Thereby, epitopes were analyzed by the VaxiJen server for antigenicity, and their scores were recorded for further analysis (Sup. file 5).

#### Linear B Lymphocytes (LBL) epitopes

With the aid of iBCE-EL server^76^, 53 LBL epitopes were predicted for S, M, N, and E proteins. 42 of them had the appropriate length of more than 6 amino acids. Out of the 42, 20 of these epitopes had been predicted to be non-toxic (by ToxinPred server) and non-allergenic (by AllerTOP 2.0 server). Like other predicted epitopes, VaxiJen scores of antigenicity were calculated for 15 predicted LBL epitopes (Sup. file 6).

### All predicted epitopes mapped back to their protein reference sequence

In order to find mutations of predicted epitopes, we mapped the location of these epitopes on their reference protein sequence. As, we have sites of mutations in proteins in “Sect. 3” section, mutations in each epitope could be identified by merging these data (Sup. Fig. S7). In this way, the number of mutations and sites of mutations in each predicted epitope was understood. As demonstrated in (Sup. Fig. S7), even the predicted epitopes have significant mutations. There is a range from 0 up to more than 200 events in some cases. As discussed before, we need to select the best ones in terms of conservity and immunogenicity for further investigation. Therefore, we continued this study with the next step and plotting the predicted data.

### Plotting the predicted epitopes with TOPSIS scoring

**Figure 8:**
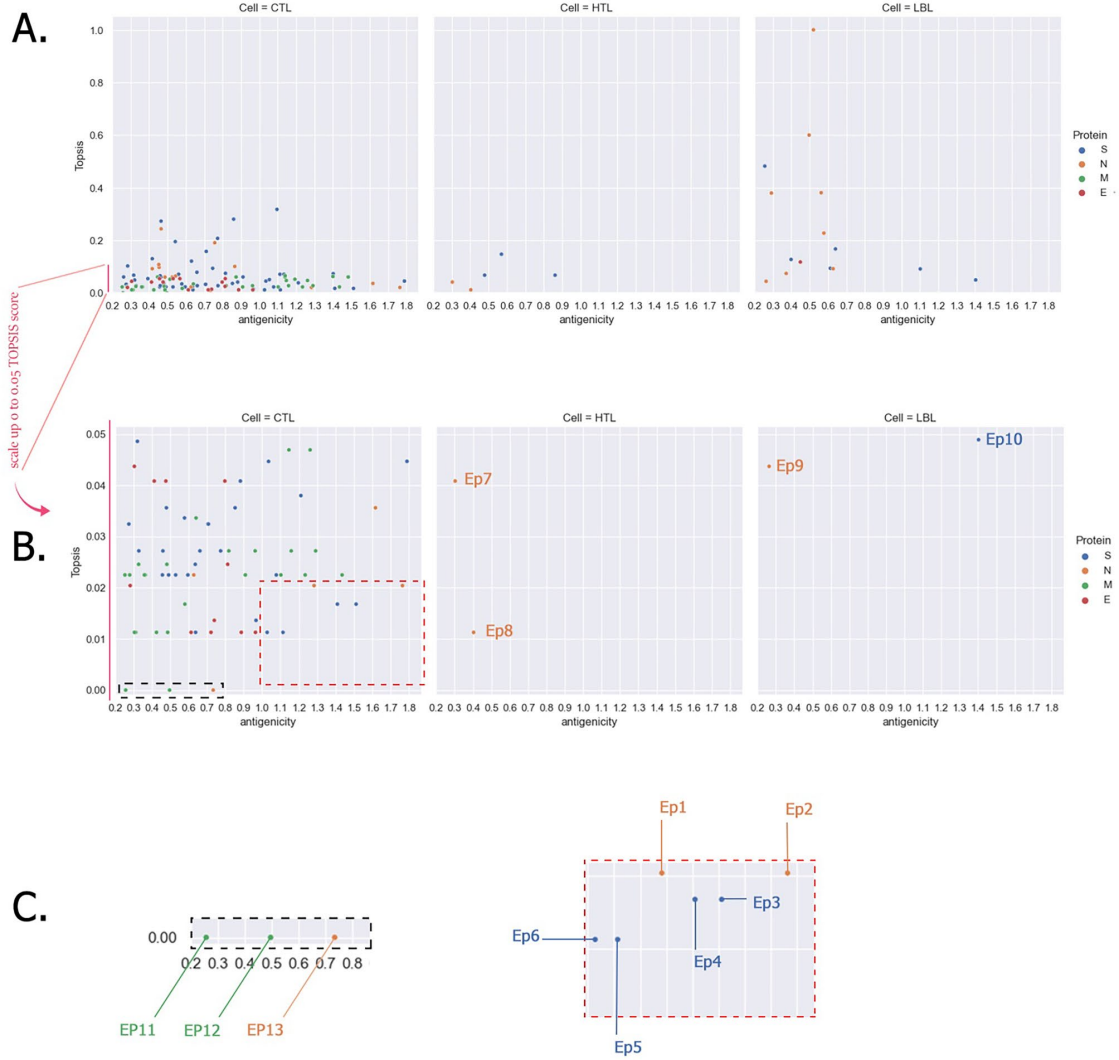
ASTP of epitopes from two perspectives. In Panel (**A**) predicted epitopes goes through a logical comparison due to TOPSIS score (vertical axis) and antigenicity (horizontal axis). This helps to differentiate between predicted epitopes with the same number of events but a distinct number of mutated sites. Again, right lower quadrant presents epitopes with higher immunologic properties besides higher conservity. Panel (**B**) scales up vertical axis (TOPSIS score) for better illustration of position of different dots (CTL, HTL, and LBL) in ASTP. The red and black boxes in CTL panel and EP7-EP10 of HTL and LBL epitopes are the most conserved ones with higher antigenicity. In panel (**C**) CTL epitopes boxes are magnified and ID of each epitope (dot) is noted. Sequence of each epitope is available in Sup. Table 4.

The Vaxijen antigenicity score of each epitope was considered as an immunological factor for the value of the Y-axis of the plot. Then, we calculated the TOPSIS scores of predicted epitopes in terms of sites and number of mutations due to Shannon entropy. In the following, the X-axis value stands for their TOPSIS score (Fig. 8 and Sup. file 7). This is clearly demonstrated in Antigenicity Score-TOPSIS Plot (ASTP) is the lower TOPSIS score in predicted epitopes in comparison with IEDB epitopes in ERTP. This may indicate that there are more appropriate options in the predicted epitopes that need further consideration. Also, proper dispersion of predicted epitopes with this method makes it easy to select the best ones based on their location in the plot. According to the plot, in the case of HTL predicted epitopes there is almost no suitable option. Either, LBL predicted epitopes offer limited options. In contrast, CTL-predicted epitopes have many appropriate choices. The closer we get to the lower right corner of the plot, the better the options.

As illustrated in Fig. 8, there are more suitable options in terms of CTL epitopes than HTL and LBL ones. In the case of HTL and LBL, there are just three epitopes with desirable properties. According to this method, the best epitopes in terms of mutation are shown in Fig. 8. Selected epitopes (Fig. 8 and Sup. Table 4) are the best epitopes ever discovered and predicted due to their immunogenicity and SARS-CoV-2 mutations.

## Discussion

Lots of mutations have been identified in the SARS-CoV-2 genome, which resulted in new variants. Potentially developing mutations can result in structural changes in key proteins involved in the pathogenesis and spread of the virus, a point that has also been shown in studies^18,20,47^. This means regardless of various approaches to vaccine design; the vaccine target needs to be selected precisely with two major points; acceptable antigenicity and appropriate conservity.

In this study, at first, we detected mutations in the SARS-CoV-2 genome by considering 3369 sequences, extracted from the GISIAD. For this purpose, first, all the available sequences were compared with the reference sequence and then the phylogenetic tree was drawn. In this way, the number of mutations and the site of them, in the variants and sequences were examined. Our approach is similar to CoVariants and Nextstrain sites to depict diversities and mutations in the genome of different viruses.

So far, several methods have been explored to find different mutations and variants. Hassan et al. and Almubaid et al. considered a method similar to what we did in finding mutations but using different software and modules^77,78^. However, other studies such as Kames et al. and another article by Hassan et al. have used other methods, by the capabilities of NCBI site and blasting tools or using MATLAB software to achieve this goal^79,80^. It was important to our study, to find the number of mutations besides the sites of mutations for further scoring and analysis. This issue needed further programming with Python to improve modules function and find custom results that are similar to Schrors et al. study^81^.

Then investigated IDRs of SARS-CoV-2 S, M, N, and E proteins have been considered for mutations. To find IDRs, IEDB immunebrowser tool was administrated with a threshold of 0.3. This approach is similar to Grifoni et al. Mukherjee et al. and S Zhuang et al. studies^82,83^.

We found that most of IDRs have mutated, just 8 of them have acceptable conservity till now. The high rate of mutations in IDRs is in accordance with several past studies but with different methods^84–87^. Schrors et al. investigated large-scale mutations in S protein and considered the variants according to diversities in this area^81^. In another study by Zhuang et al. also, the IDRs mutations located in S protein were investigated for mutations^83^. A large number of mutations in both last articles is acceptable according to our result^81,83^. Until now, IDRs have not been extensively and comprehensively examined for mutations in SARS-CoV-2. In this study for the first time, all of these areas were examined with a comprehensive scientific approach. Furthermore, IDRs were plotted with normalized factors to compare these areas, which had not been addressed before. This plot simplifies the selection of the best IDRs to target.

Furthermore, we presented evidence of significant mutations in B cell linear epitopes and HTL and CTL epitopes from S, M, N, and E proteins of SARS-CoV-2, which are discussed in peer reviews extracted from IEDB. Mutations in epitopes have also been presented in other studies^88,89^. In previous studies, only one or several epitopes were examined for mutations, but in this study, with a comprehensive view, all IEDB epitopes were examined for this purpose.

Finding mutation itself is not enough to find the best epitopes. Thereupon in a step beyond, we scored their events with the TOPSIS method to quantify their conserved manner. In the setting of this algorithm and plotting with their immunogenicity properties the best epitopes were selected for further analysis in the laboratory. In Mullick et al. study, they investigated S protein mutations hotspot through Shannon entropy and K-means clustering^90^. Their approach in terms of scoring is different from ours, but their efforts to find a suitable way to score areas with high mutations as hotspots are admirable.

As our results showed, almost all epitopes have changed through mutations, which challenge the immune response against SARS-CoV-2 over time. This could also explain the generation of new and more dangerous variants or VOCs. In Garrett et al. study, S protein was investigated through variants with the Phage-DMS approach in the laboratory. They have shown that antibody neutralization has also changed as a result of IDRs mutation^91^. This confirms our hypotheses in this article in terms of the huge number of mutations in IDRs and epitopes that challenge the immune system and the importance of finding conserve epitopes to target.

There are several different scoring methods. Here we needed to weigh each factor (number of mutations and sites of mutations) in terms of conservity unequally with Shannon entropy. In this case, TOPSIS was preferred as the best choice for further scoring.

Also, we have tested this approach (TOPSIS scoring and plotting) in predicted epitopes. To predict the epitopes there are different databases and approaches. In this article, we have predicted epitopes with a platform designed in Ahammad et al. study^92^. After prediction of epitopes, we ran Shannon entropy and TOPSIS scoring and plotting through antigenicity and conservity, successfully for identification of best epitopes with two factors. In addition, having a quantitative view in terms of conservity and antigenicity with efficient illustration and plotting can help to simplify rational decision-making in a variety of epitopes.

The next and final section was to select the best epitopes. Illustration of epitopes scores (by plotting) helps to understand their different properties on a large scale. Besides, having a quantitative measurement in terms of conservity can help to make rational decisions. This plot provides a roadmap understanding antigens and epitopes alteration in variants. Also, this could help to select epitopes by considering different criteria. This method is an open-source multi-criteria platform for deciding on the best epitopes.

Our findings indicated the rapid and significant changes in IDRs and epitopes. This highlights the importance of a system for the efficient development of vaccines against new strains and variants. As this platform retrospectively finds previous changes and mutations in epitopes and IDRs, it could help to understand the development of recent emerging variants.

### The limitation of our study

There are two main categories of epitopes: linear epitopes and conformational or 3 d epitopes. T cells recognize linear epitopes and B cells can recognize conformational epitopes and linear ones^93–95^. As we aimed to understand changes in the sequence of proteins, we focused on linear epitopes of IEDB and predicted ones. Of course, mutations can make conformational changes in epitopes and antigens tertiary structure and affects B cells' recognition of epitopes. This issue (considering conformational changes in epitopes with mutations) is important to be addressed by further research.

In this study, we faced with the limitation of computing power, so instead of using all the genomes found in the world, we tried to use those that have the most differences and located in different components of the phylogeny tree. Although this problem has been solved with a scientific and algorithmic approach (Sup. Algorithm 1), it should be taken into consideration by other researchers.

Our platform is not able to predict future variations and it is based on past mutations that happened in the SARS-CoV-2 genome. It will help to understand mutations retrospectively and select appropriate epitopes against recent emerging variants. This platform needs recent new variants’ sequences data to be up to date for further analysis.

As we aimed to find a method for the selection of epitopes with different properties, we didn’t focus on vaccine design. In the end, we just mention several epitopes as a potential potential candidate for vaccine design. Of course, these epitopes need further investigation like; vaccine constructs design, molecular docking, prediction of population coverage, and laboratory studies.

## Conclusion

As we face a pandemic caused by an ever-changing virus; providing a scientific and practical approach to select epitopes with appropriate conservity and immunogenicity seems to be crucial.

Also, we found some predicted epitopes are more conserved and immunogen as a potential candidate for vaccine design (Sup. Table 4). According to our findings (Sup. Table 4 and Fig. 8), LSPRWYFYY and DLSPRWYFY predicted CTL epitopes from N protein, and VVFLHVTYV, GVVFLHVTY, VRFPNITNL, and PYRVVVLSF predicted CTL epitopes from S protein are highly conserved and immunogen and suitable for vaccine design. Using an AAY linker between CTL epitopes, we can design a multiple-epitope vaccine. Also, WPQIAQFAPSASAFF and QIAQFAPSASAFFGM predicted HTL epitopes from N protein and AGLPYGANK predicted LBL epitopes from N protein can be added to CTL epitopes through linkers (like GPGPG). There are other choices for vaccine design between predicted epitopes and IEDB epitopes that can be added to this vaccine. Here we selected the 7 best epitopes for vaccine construct design.

At the end, we introduce a scientific method, with the hope that this protocol will aid in the development of vaccine design against SARS-CoV-2, particularly the VOC.

## Methods

### Detecting site and number of mutations in variants

In order to detect mutations made over the past year in the SARS-CoV-2 genome, the following procedure was done (Fig. 1). First, SARS-CoV-2 complete genome sequences have been collected from the global initiative on sharing avian flu data (GISAID) database (http://www.gisaid.org). Then, the collected sequences were aligned with the reference genome (hCoV-19/Wuhan/Hu-1/2019) by Multiple Alignment using Fast Fourier Transform [MAFFT; a multiple sequence alignment program (cbrc.jp)]. In the following, sequences with more than 3000 not read bases (Ns) and gaps (‘–’), wrong dates before 2019, and sequences shorter than 1000 base pairs (bp) were excluded from the analysis.

Herein, using IQ-TREE^56^ [Cibiv/IQ-TREE: Efficient phylogenomic software by maximum likelihood (github.com)] phylogenetic tree was constructed for all remaining sequences. In the next step phylogenetic tree needed to reroot and resolve polytomies. Therefore, TreeTime [neherlab/treetime: Maximum likelihood inference of time stamped phylogenies and ancestral reconstruction (github.com)] infers the tree’s internal nodes dates and prone sequences.

Afterward, using the augur Ancestral module [augur/ancestral.py at master next strain/augur (github.com)], mutations in genomes, and the phylogenetic tree’s internal nodes were inferred in their sequence.

In order to translate sequences and their mutations into Amino Acids, we used the augur translate module [augur/translate.py at master nextstrain/augur (github.com)]. Employing predefined Amino acids mutations (mutations in protein sequence) as input, we calculated the diversity of each SARS-CoV-2 genome region by Python programming (version 3.8).

### Exploring mutations within IDRs of SARS-CoV-2

B cell, HTL, and CTL epitopes from S, M, N, and E proteins were extracted from IEDB. Then search result was restricted to epitopes with recognition by human leukocyte antigen (HLA) that is the human version of MHC molecules, In the following, extracted epitopes were mapped back to a SARS-CoV-2 reference sequence^53^ using the IEDB’s immunobrowser tool^57^. As all available records aligned along the reference sequence, the RF score was calculated by the positivity rate (positive response noted) divided by the total number of records (number of independent assays) (see the following equation) ^58^.

$${\text{RF}} = {\text{ Positive response rate}}/{\text{Total number of records}}$$

In this way, IDRs were identified by considering their RF score or lower bound in the diagram (where the RF score was RF ≥ 0.3 considered as IDR) (Fig. 4, Sup. Figs. 2.1 and 2.2).

### Discovering mutations in SARS-CoV-2 IEDB epitopes

Extracted B cell, HTL, and CTL epitopes of M, N, E, and S proteins from IEDB were aligned to their reference sequence, in order to map the mutational diagram of each protein. As we have the number of events and mutations in each site of the reference sequence in step 2, we could find out where each epitope is located in its protein sequence and how this located part has undergone alterations by mutations.

### Plotting events against immunologic properties of IEDB epitopes

We chose RF of IEDB epitopes as the X-axis value. Besides, all mutations (without considering the site of mutation) in each epitope have been selected as events of epitopes for the Y-axis. In this way EVRP clearly depicts past events to each epitope besides their immunologic property.

### Epitope prediction

#### CTL epitope prediction

NetCTL v1.2 server is a powerful free source for predicting CTL epitopes^71^. This server integrates the prediction score of MHC class I binding peptides and; proteasomal C terminal cleavage with transporter associated with antigen processing (TAP) transport efficiency score; to deliver an integrated score for CTL epitope prediction from a sequence. In this study, 9-mer CTL epitopes were predicted by using the NetCTL v1.2 server for 12 MHC class I supertypes (A1, A2, A3, A24, A26, B7, B8, B27, B39, B44, B58, and B62)^72^. Epitopes that reached above 0.75 scores as threshold was selected for the next step^92^. There is no reference and definite value for thresholds. Thereupon, we followed other articles used similar methods to find the value of threshold (> 0.75)^92,96,97^.

To predict the immunogenicity of the CTL epitopes, the Class I immunogenicity tool of the IEDB Analysis Resource was administrated^40^. Epitopes with a positive value for immunogenicity were selected for the next steps (A percentile rank score ≤ 2).

Here is to note that there is no reference value for the percentile rank. According to similar studies and our study design, percentile rank ≤ 0.25 was considered for this study^92,98^.

#### HTL epitope prediction

To predict 15-mer HTL epitopes and their MHC class II alleles, a consensus algorithm of the IEDB MHC class II binding tool was administrated^41,99,100^. Epitopes with percentile rank ≤ 0.25 were considered for the next steps. It is important to note that there is no reference value for the percentile rank. According to similar studies and our study design, percentile rank ≤ 0.25 was considered for this study^92,98^.

#### Prediction of linear B cell epitopes

For the prediction of Linear B cell epitopes iBCE-EL server was used^76,101^. Epitopes with positive values were selected for further analysis.

#### Allergenicity prediction

Allergenicity of epitopes was assessed using AllerTOP 2.0 server^70^. The non-allergic epitopes were subjected to the next steps for further analysis.

#### Toxicity prediction

The toxicity of selected epitopes was evaluated using the ToxinPred server^102,103^. The non-toxic epitopes were subjected to the next steps for further analysis.

### TOPSIS scoring method and Shannon entropy

TOPSIS is one of the best multiple decision-making methods. In this method, ’i’ is the number of alternatives that can be evaluated by the number of attributes ‘j’. In the decision matrix, (epitopes) were considered as alternatives (the total number of mutations), and (the number of sites of mutations) in each epitope was considered as attributes. Attributes were weighted by Shannon entropy. Then, the decision matrix was normalized. The normalized decision matrix (N) was multiplied by a diagonal matrix of attributes weights (Wj × j). The positive ideal solution (Vj+) and negative ideal solution (Vj−) were determined by a weighted normalized decision matrix (V). The difference between each attribute of epitopes from positive and negative ideal solutions (Vj, Vj−) was calculated. The relative closeness of each epitope to the ideal solution was determined. By sorting epitopes into rating order, the epitopes with fewer scores were detected as more conserve ones. It should be mentioned that the epitope, which gets a score of zero in the TOPSIS method, may not be absolutely conserved. The score of zero stands for more conserved epitopes than the other evaluated epitopes. TOPSIS matrix equation and Shannon entropy equation are noted in the supplementary file equation part.

### Predicted epitopes events and plotting with TOPSIS

We mapped the location of predicted epitopes on their reference protein sequence, in order to find mutations of predicted epitopes. As we have sites of mutations in proteins in past sections, mutations in each predicted epitope could be identified by merging these data.

The Vaxijen antigenicity score of each epitope was considered as the value of the Y-axis of the plot. Then, we calculated the TOPSIS scores of predicted epitopes in terms of sites and number of mutations. In the following, the Y-axis value stands for their TOPSIS score for plotting. The same has been done for IEDB epitopes.

### Ethics approval

No human or animal models were utilized in this investigation. All experiments were performed according to the guidelines of the Medical Ethics Committee of the Jahrom University of Medical Sciences (IR.JUMS.REC.1399.090).

## Supplementary Information

Supplementary Information 1.

Supplementary Information 2.

Supplementary Information 3.

Supplementary Information 4.

Supplementary Information 5.

Supplementary Information 6.

Supplementary Information 7.

## Data Availability

Our data and codes are available through corresponding author if asked.
