# Supplementary material for: Considering epitopes conservity in targeting SARS-CoV-2 mutations in variants: a novel immunoinformatics approach to vaccine design
Source: Sci Rep. 2022 Aug 18;12:14017. doi: 10.1038/s41598-022-18152-5 (PMC9386201; doi:10.1038/s41598-022-18152-5)
Supplement: Supplementary file 1 — Supplementary Information 1. [file 41598_2022_18152_MOESM1_ESM.docx]

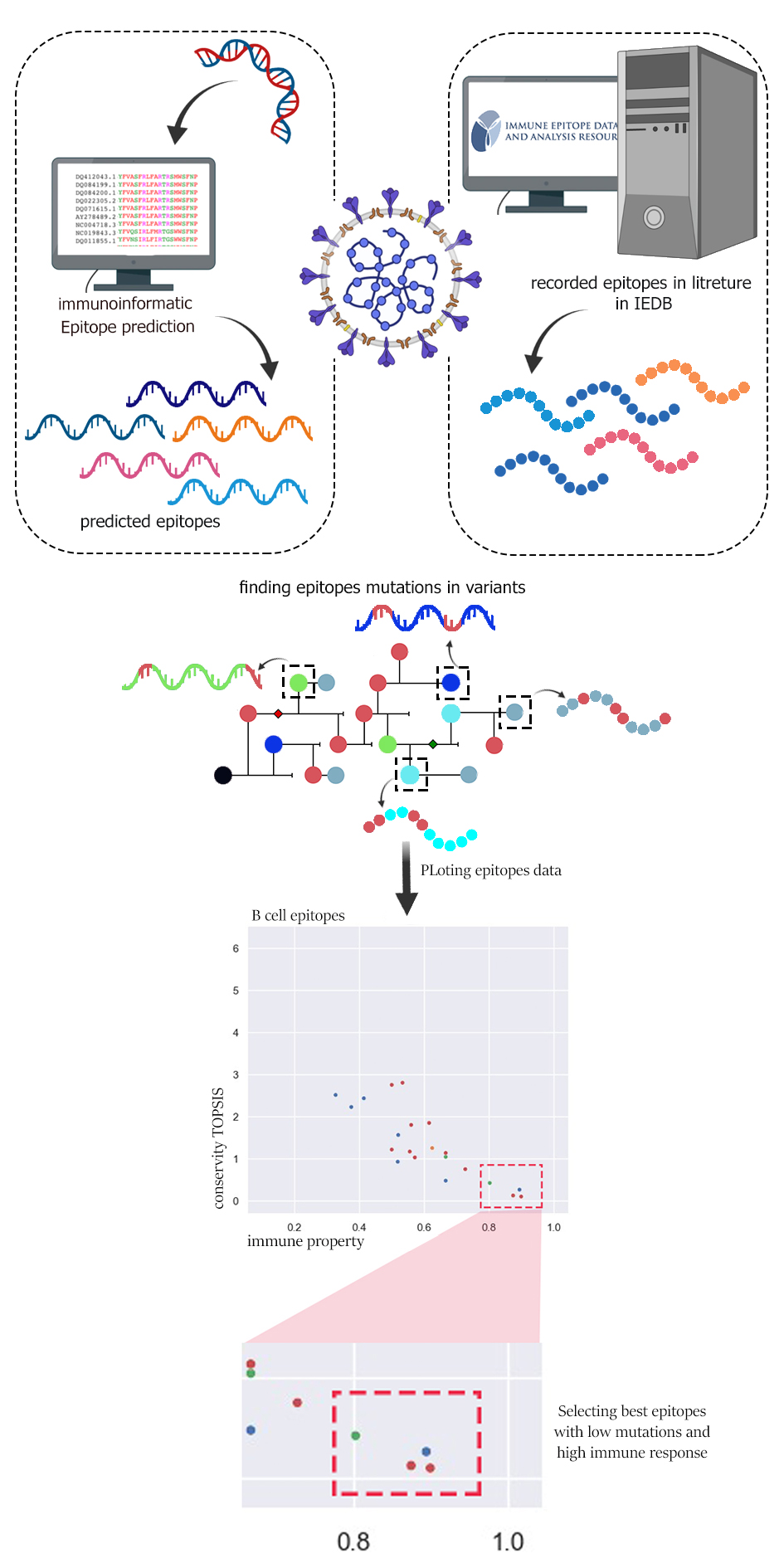


**sup diagram 1 – study design**


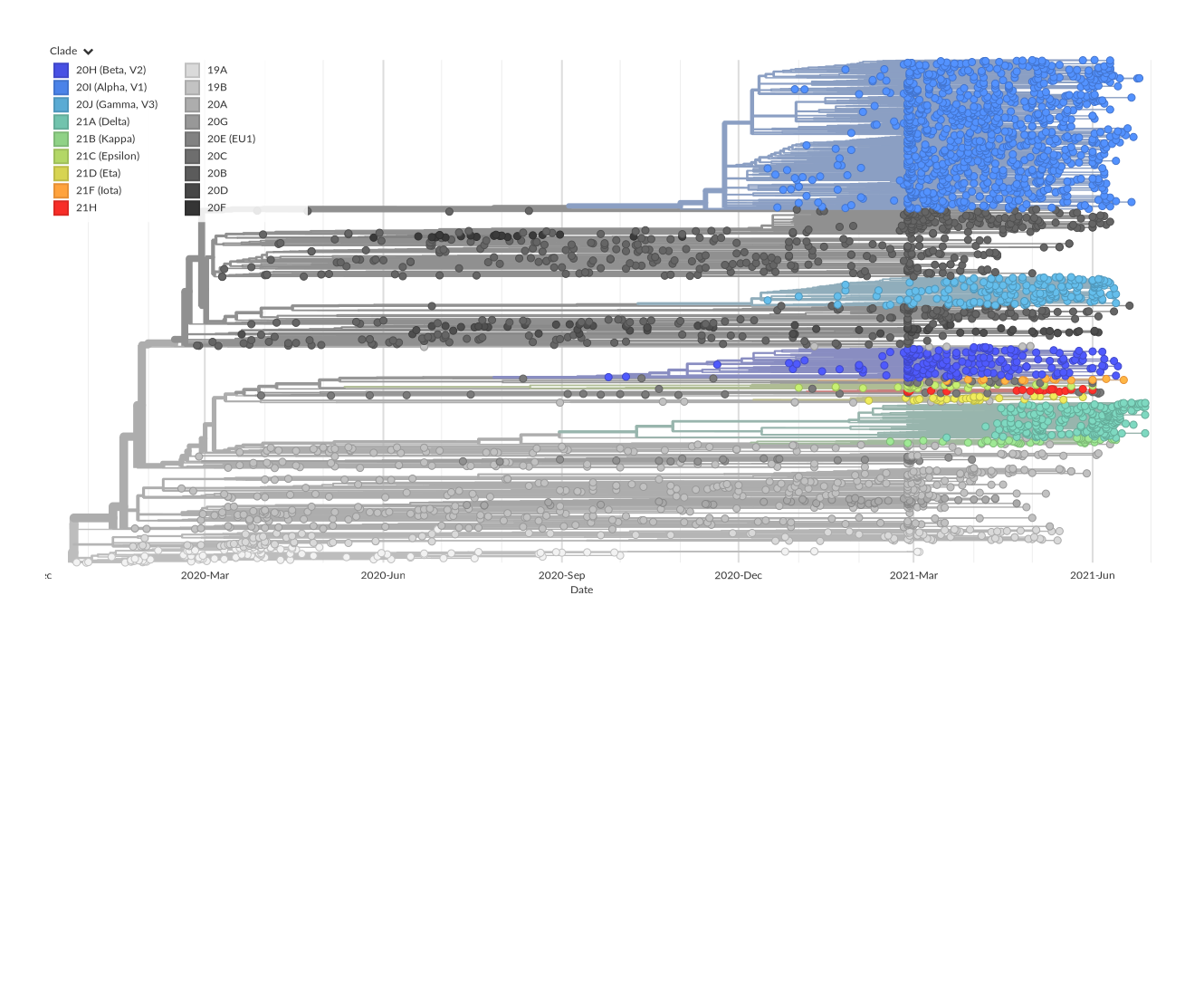


**Sup fig 1. Phylogenetic tree of SARS-COV-2**


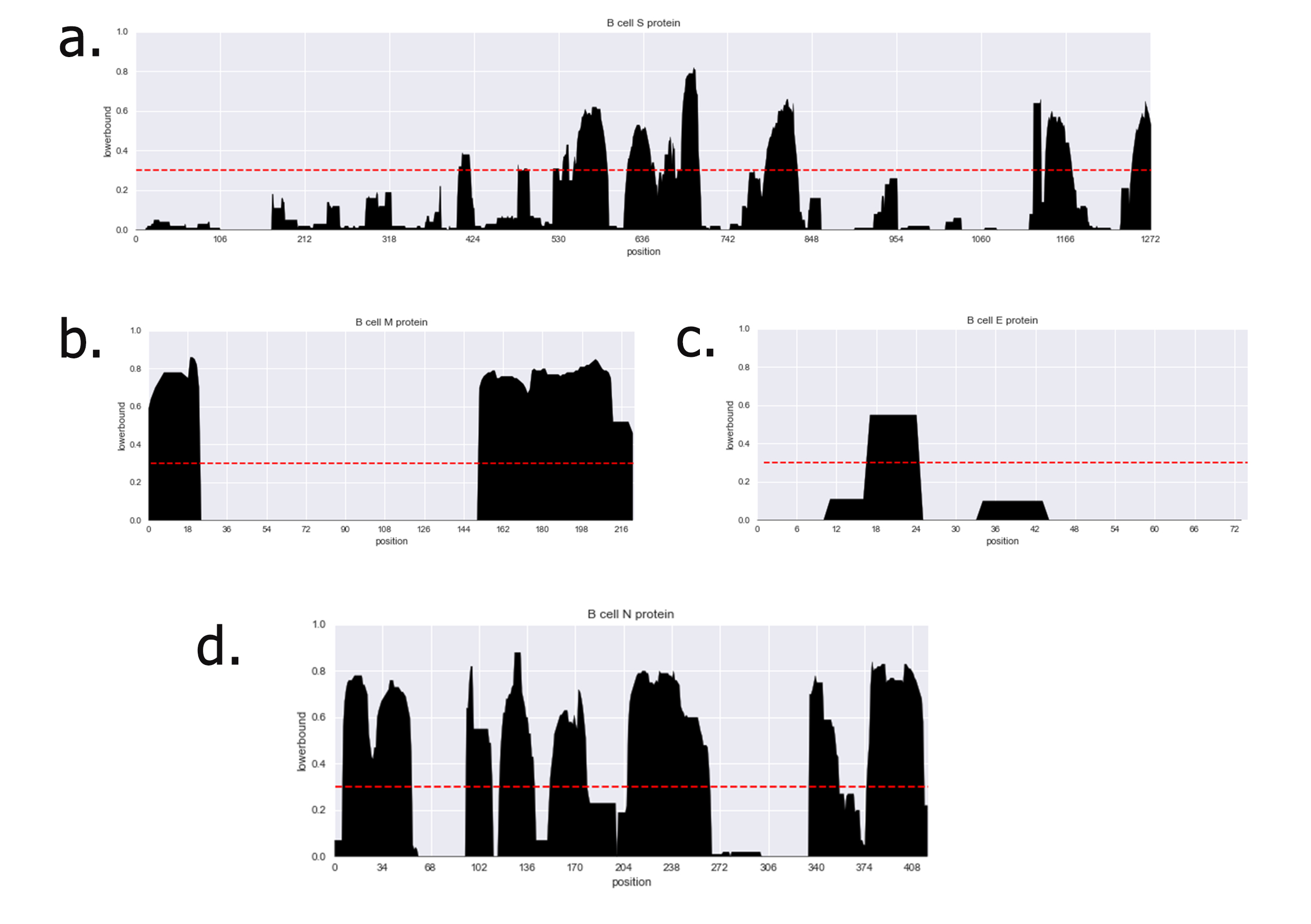


**Sup Fig 2. B cell IDRs of SARS-CoV-2 surface proteins are calculated and demonstrated through lower bound level threshold.** B cell IDRs of S protein illustrated in sup fig 2.a; B cell IDRs of M protein illustrated in sup fig 2.b; B cell IDRs of E protein illustrated in sup fig 2.c; B cell IDRs of N protein illustrated in sup fig 2.d.


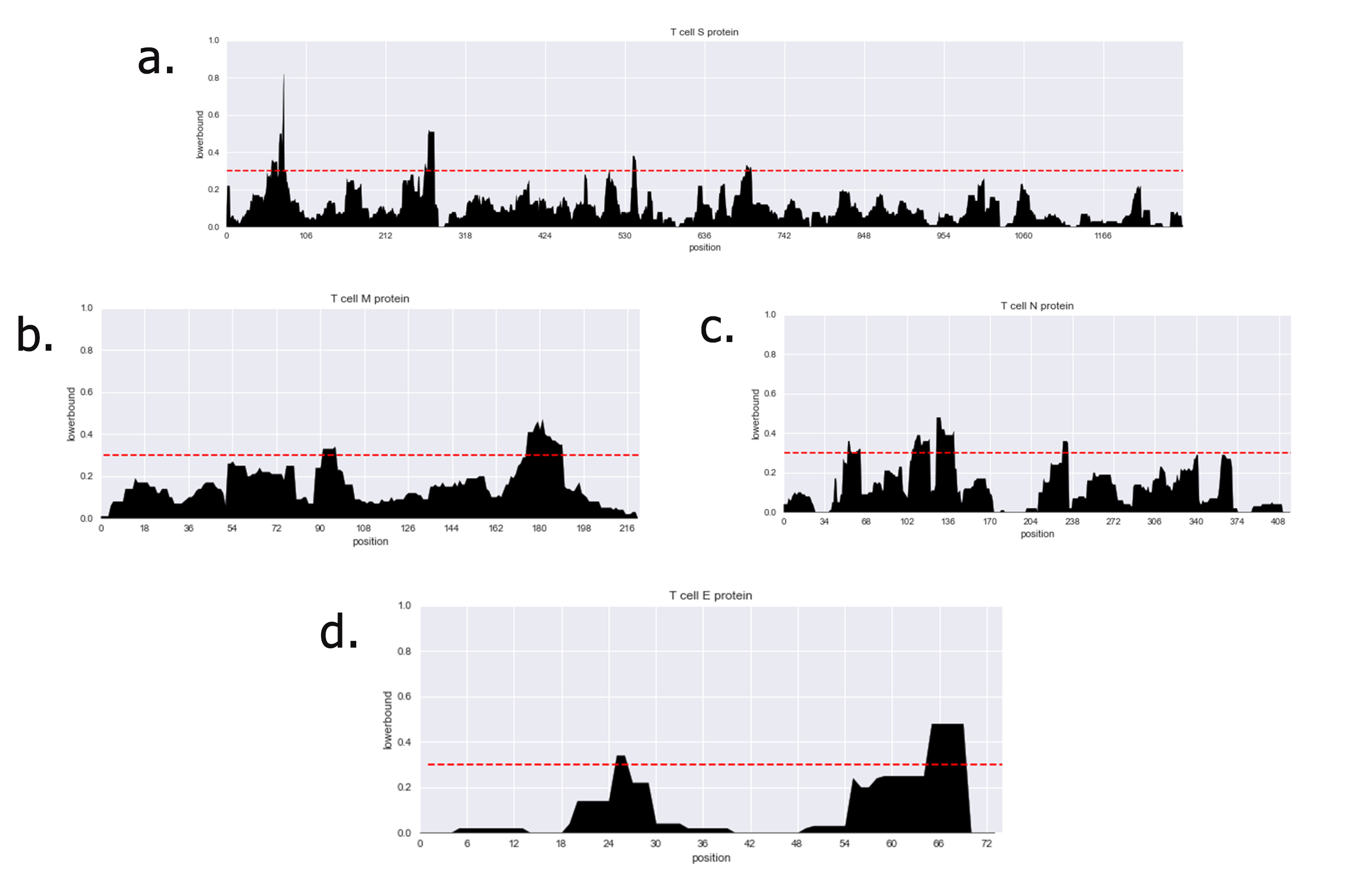


**Sup Fig 3. T cell IDRs of SARS-CoV-2 surface proteins are calculated and demonstrated through lower bound level threshold.** T cell IDRs of S protein illustrated in sup fig 3.a; T cell IDRs of M protein illustrated in sup fig 3.b; T cell IDRs of N protein illustrated in sup fig 3.c; T cell IDRs of E protein illustrated in sup fig 3.d.

| N pro B cell | L_IDR | NCS | NMS | NE | NE/L_IDR | NCS/L_IDR |
| --- | --- | --- | --- | --- | --- | --- |
| IDR1 | 49 | 16 | 33 | 123 | 2.510204 | 0.326531 |
| IDR2 | 19 | 17 | 2 | 5 | 0.263158 | 0.894737 |
| IDR3 | 25 | 13 | 12 | 39 | 1.56 | 0.52 |
| IDR4 | 27 | 14 | 13 | 25 | 0.925926 | 0.518519 |
| IDR5 | 58 | 24 | 34 | 141 | 2.431034 | 0.413793 |
| IDR6 | 21 | 14 | 7 | 10 | 0.47619 | 0.666667 |
| IDR7 | 40 | 15 | 25 | 89 | 2.225 | 0.375 |

S. Table 1.a

| E pro B cell | L_IDR | NCS | NMS | NE | NE/L_IDR | NCS/L_IDR |
| --- | --- | --- | --- | --- | --- | --- |
| IDR1 | 8 | 5 | 3 | 10 | 1.25 | 0.625 |

S. Table 1.b

| M pro B cell | L_IDR | NCS | NMS | NE | NE/L_IDR | NCS/L_IDR |
| --- | --- | --- | --- | --- | --- | --- |
| IDR1 | 24 | 16 | 8 | 25 | 1.041667 | 0.666667 |
| IDR2 | 71 | 57 | 14 | 30 | 0.422535 | 0.802817 |

S. Table 1.c

| S pro B cell | L_IDR | NCS | NMS | NE | NE/L_IDR | NCS/L_IDR |
| --- | --- | --- | --- | --- | --- | --- |
| IDR1 | 15 | 10 | 5 | 17 | 1.133333 | 0.666667 |
| IDR2 | 15 | 8 | 7 | 42 | 2.8 | 0.533333 |
| IDR3 | 8 | 7 | 1 | 1 | 0.125 | 0.875 |
| IDR4 | 8 | 7 | 1 | 1 | 0.125 | 0.875 |
| IDR5 | 44 | 32 | 12 | 33 | 0.75 | 0.727273 |
| IDR6 | 35 | 20 | 15 | 36 | 1.028571 | 0.571429 |
| IDR7 | 13 | 8 | 5 | 24 | 1.846154 | 0.615385 |
| IDR8 | 28 | 14 | 0 | 77 | 2.75 | 0.5 |
| IDR9 | 36 | 20 | 0 | 42 | 1.166667 | 0.555556 |
| IDR10 | 10 | 9 | 1 | 1 | 0.1 | 0.9 |
| IDR11 | 28 | 14 | 0 | 34 | 1.214286 | 0.5 |
| IDR12 | 25 | 14 | 0 | 45 | 1.8 | 0.56 |

S. Table 1.d

**S. Table 1- B cell IDRs calculated scores are presented in these tables.** N protein B cell IDRs calculated scores are presented in S. Table 1.a. E protein B cell IDRs calculated scores are presented in S. Table 1.b. M protein B cell IDRs calculated scores are presented in S. Table 1.c. S protein B cell IDRs calculated scores are presented in S. Table 1.d.

| E pro T cell | L_IDR | NCS | NMS | NE | NE/L_IDR | NCS/L_IDR |
| --- | --- | --- | --- | --- | --- | --- |
| IDR 1 | 2 | 2 | 0 | 0 | 0 | 1 |
| IDR 2 | 5 | 2 | 3 | 8 | 1.6 | 0.4 |

S. Table 2.a

| M pro T cell | L_IDR | NCS | NMS | NE | NE/L_IDR | NCS/L_IDR |
| --- | --- | --- | --- | --- | --- | --- |
| IDR 1 | 6 | 6 | 0 | 0 | 0 | 1 |
| IDR 2 | 15 | 10 | 5 | 6 | 0.4 | 0.666667 |

S. Table 2.b

| N pro T cell | L_IDR | NCS | NMS | NE | NE/L_IDR | NCS/L_IDR |
| --- | --- | --- | --- | --- | --- | --- |
| IDR 1 | 11 | 10 | 1 | 9 | 0.818182 | 0.909091 |
| IDR 2 | 15 | 13 | 2 | 6 | 0.4 | 0.866667 |
| IDR 3 | 15 | 7 | 8 | 32 | 2.133333 | 0.466667 |
| IDR 4 | 5 | 2 | 3 | 31 | 6.2 | 0.4 |

S. Table 2.c

| S pro T cell | L_IDR | NCS | NMS | NE | NE/L_IDR | NCS/L_IDR |
| --- | --- | --- | --- | --- | --- | --- |
| IDR 1 | 8 | 3 | 5 | 17 | 2.125 | 0.375 |
| IDR 2 | 10 | 1 | 9 | 32 | 3.2 | 0.1 |
| IDR 3 | 13 | 7 | 6 | 6 | 0.461538 | 0.538462 |
| IDR 4 | 5 | 5 | 0 | 0 | 0 | 1 |
| IDR 5 | 8 | 7 | 1 | 1 | 0.125 | 0.875 |

S. Table 2.d

**S. Table 2. T cell IDRs calculated scores are presented in these tables.** E protein T cell IDRs calculated scores are presented in S. Table 2.a. M protein T cell IDRs calculated scores are presented in S. Table 2.b. N protein T cell IDRs calculated scores are presented in S. Table 2.c. S protein T cell IDRs calculated scores are presented in S. Table 2.d.

| Events in sites of proteins | | |  |  |
| --- | --- | --- | --- | --- |
| Protein | Number of sites of Amino acids in protein | Number of sites with at least on event | Number of conserve sites | Total number of events in the protein |
| S | 1273 | 454 (35%) | 819 | 1670 |
| E | 75 | 23 (30%) | 52 | 64 |
| M | 222 | 60 (27%) | 162 | 132 |
| N | 419 | 207 (49%) | 212 | 787 |

**S.Table 3. Events in sites of proteins**


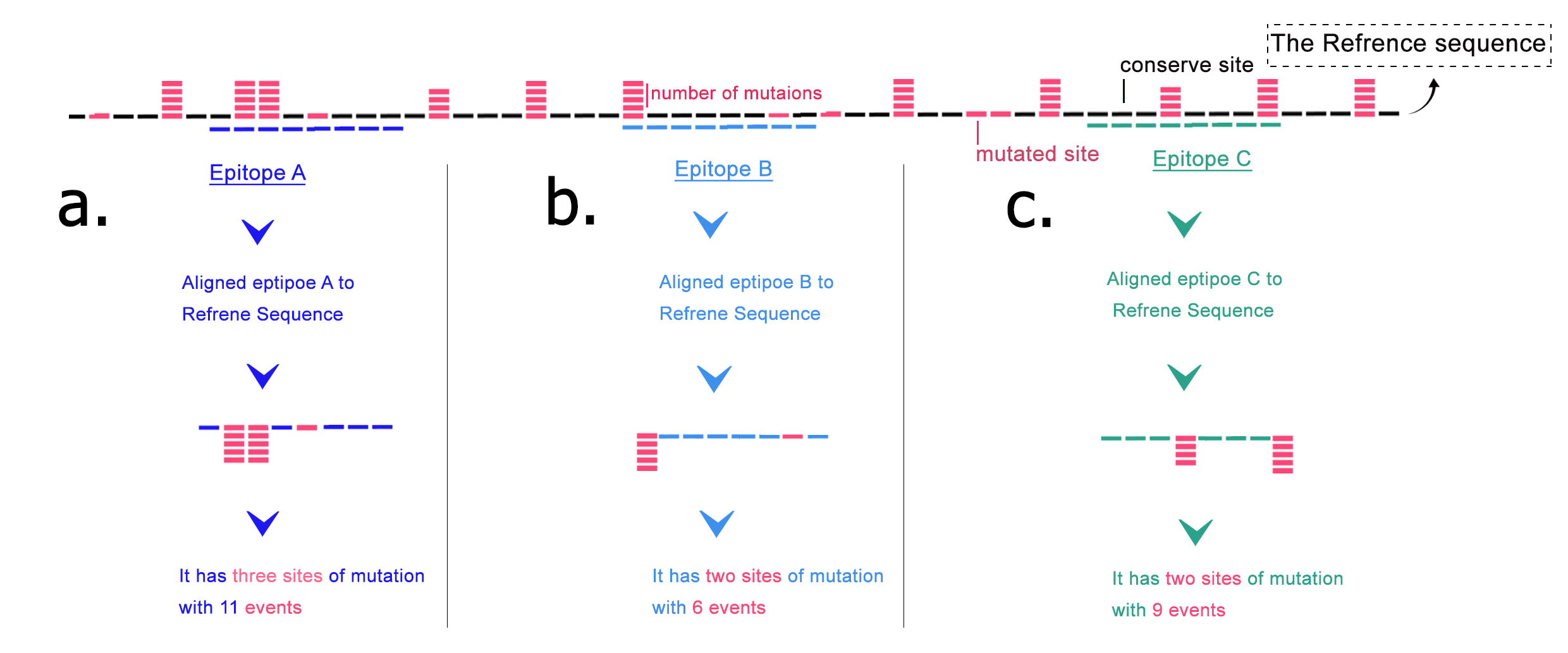


**Sup fig.4. Schematic illustration of IEDB epitopes mutation extraction from reference sequence;** epitopes where align back to reference genome to find sites of mutations in epitopes and number of their events. In panel a. there is an epitope with 3 sites of mutations and 11 events in comparison with reference sequence. In panel b. there is an epitope with 2 sites of mutations and 6 events in comparison with reference sequence. In panel c. there is an epitope with 2 sites of mutations and 9 events in comparison with reference sequence.


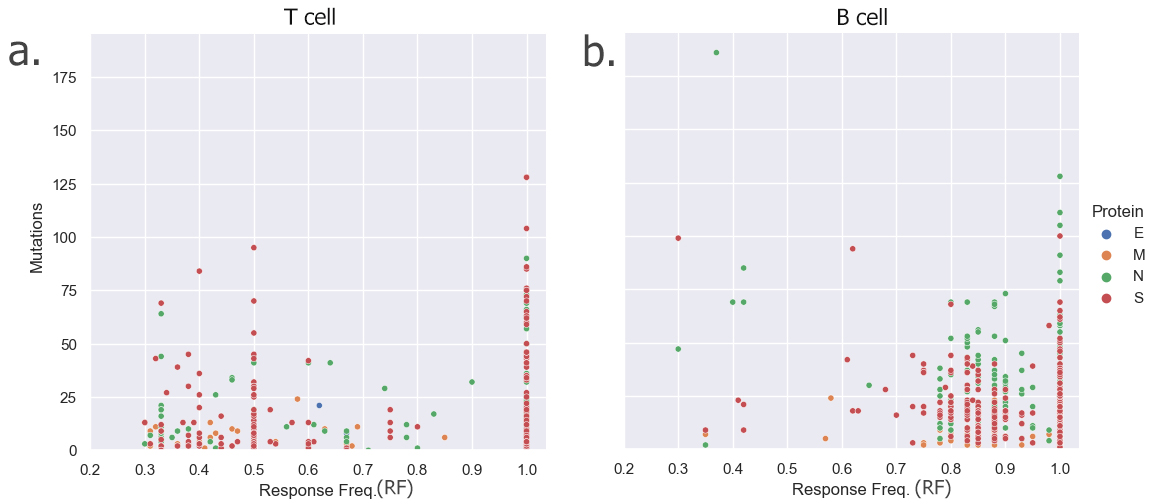


**Sup fig.5. EVRP illustrates IEDB B cell (in the Left) and T cell (in the Right) epitopes immunologic property (by RF) along with their number of mutations**. Obviously, the epitopes located at the bottom and rights of the plot are suitable for both features. In panel a. IEDB T cell epitopes are plotted and in panel b. IEDB B cell epitopes are plotted with EVRP.


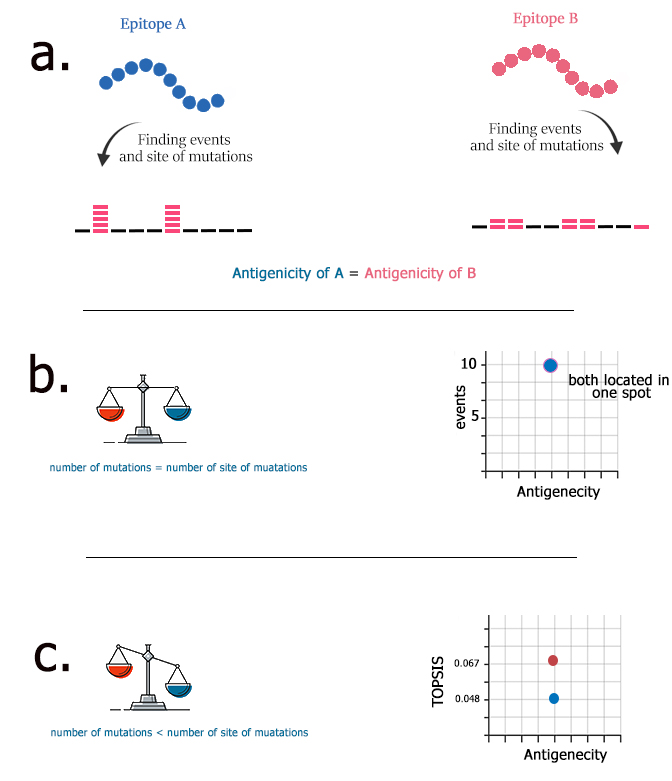


**Sup fig.6. Schematic comparison of TOPSIS scoring vs events.** The same weighted factors (panel b) against unequal weighting the factors due to Shannon entropy and TOPSIS result for decision making (panel c). In panel a. two epitopes with the same antigenicity and number of mutations but different mutated sites. In panel b both epitopes are compared with the same weight of sites of mutation and number of mutations. Therefore both epitopes are located in one spot in the plot (have no difference with this method). In another way, in panel c. the weight of the site of mutations is more than the number of mutations with TOPSIS calculation. Therefore, the difference between the two epitopes becomes prominent in the plot.


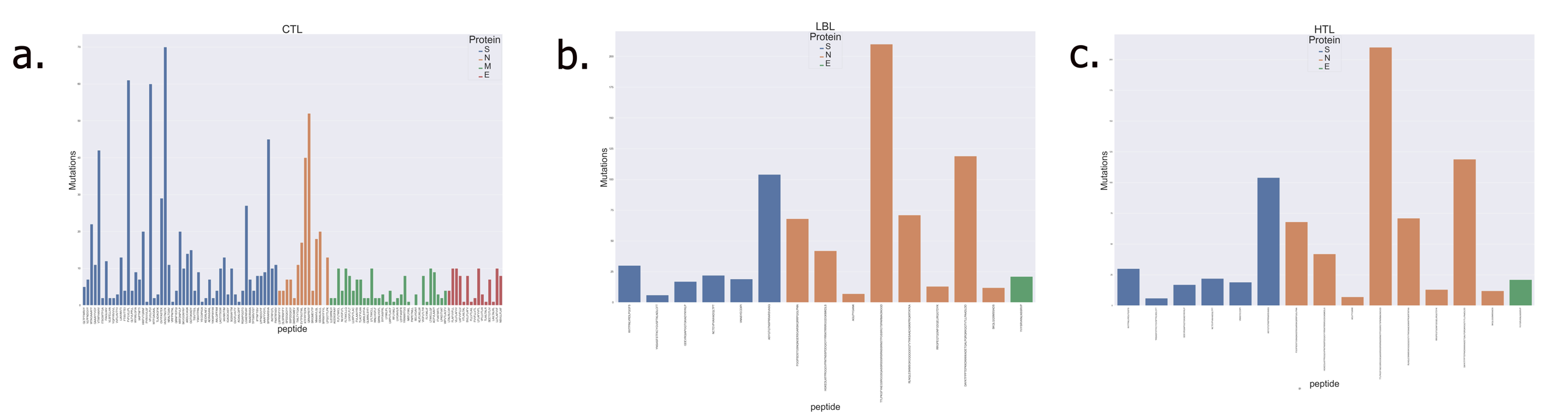


**Sup fig.7. Events in predicted epitopes.** As illustrated, there is diversity in terms of mutations in different epitopes. As mentioned the sequence of each epitope is noted in X axis. Y axis is present for number of mutations. In panel a. CTL predicted epitopes numbers of mutations are illustrated. In panel b. LBL predicted epitopes numbers of mutations are illustrated. Also, in panel c. LBL predicted epitopes numbers of mutations are illustrated.

| Epitope ID | Peptide | Protein | Antigenicity | TOPSIS |
| --- | --- | --- | --- | --- |
| EP1 | LSPRWYFYY | N | 1.2832 | 0.020434 |
| EP2 | DLSPRWYFY | N | 1.7645 | 0.020434 |
| EP3 | VVFLHVTYV | S | 1.5122 | 0.016803 |
| EP4 | GVVFLHVTY | S | 1.4104 | 0.016803 |
| EP5 | VRFPNITNL | S | 1.1141 | 0.011294 |
| EP6 | PYRVVVLSF | S | 1.0281 | 0.011294 |
| EP7 | WPQIAQFAPSASAFF | N | 0.3028 | 0.04086 |
| EP8 | QIAQFAPSASAFFGM | N | 0.4032 | 0.011294 |
| EP9 | AGLPYGANK | N | 0.2631 | 0.043741 |
| EP10 | YNSASFSTFKCYGVSPTKLNDLCFT | S | 1.4031 | 0.048938 |
| EP11 | FNPETNILL | M | 0.2578 | 0 |
| EP12 | KLIFLWLLW | M | 0.4968 | 0 |
| EP13 | SPRWYFYYL | N | 0.734 | 0 |

**S.Table 4. Sequence of selected epitopes.**

**Equations:**


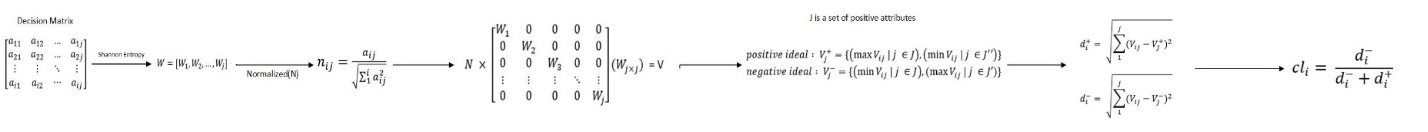
**TOPSIS matrix and equation.**


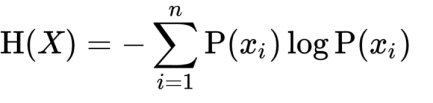


**Shannon entropy equation. Xi represents for possible outcomes and P(Xi) stands for the probability of Xi. H(X) is the entropy of variable X.**

**Sup. Algorithm 1.** This demonstrates how sequences were selected from GISAID.
